# *Streptococcus suis* Stk1 sensitizes epithelial cells to ferroptosis and exacerbates disruption of the respiratory epithelial barrier

**DOI:** 10.1080/22221751.2026.2627066

**Published:** 2026-02-02

**Authors:** Lang Tian, Ruicheng Yang, Ting Qi, Wenquan Ouyang, Hongshuo Liu, Dong Huo, Hang Li, Chuyue Zhou, Manman Xu, Haojie Li, Qingyun Liu, Dang Wang, Chen Tan, Huanchun Chen, Xiangru Wang

**Affiliations:** a National Key Laboratory of Agricultural Microbiology, College of Veterinary Medicine, Huazhong Agricultural University, Wuhan, People’s Republic of China; b Key Laboratory of Preventive Veterinary Medicine in Hubei Province, the Cooperative Innovation Center for Sustainable Pig Production, Wuhan, People’s Republic of China; c Engineering Research Center of Animal Biopharmaceuticals, The Ministry of Education of the People's Republic of China (MOE), Wuhan, People’s Republic of China; d Frontiers Science Center for Animal Breeding and Sustainable Production, Huazhong Agricultural University, Wuhan, People’s Republic of China; e Institute of Microalgae Synthetic Biology and Green Manufacturing, School of Life Sciences, Jianghan University, Wuhan, People’s Republic of China

**Keywords:** *Streptococcus suis*, ferroptosis, respiratory epithelial barrier, Stk1, Nrf2

## Abstract

*Streptococcus suis* serotype 2 (SS2), a significant zoonotic pathogen, initiates systemic infection by breaching the respiratory epithelial barrier. Ferroptosis, an iron-dependent form of regulated cell death driven by lipid peroxidation, is increasingly implicated in the pathogenesis of various infectious diseases, yet its role in SS2-induced epithelial barrier dysfunction remains unknown. Here, we demonstrate SS2 infection sensitizes airway epithelial cells to ferroptosis, leading to the accumulation of lipid peroxides, upregulation of the transcriptional repressor Snail1, and subsequent downregulation of intercellular junction proteins. This cascade compromises epithelial integrity and promotes bacterial translocation. Mechanistically, we found SS2 overwhelms the cellular redox defense system and identified bacterial eukaryotic-like serine/threonine kinase 1 (Stk1) as the key mediator of this process. Stk1 directly interacts with host protein Keap1, which stabilizes the Keap1-Nrf2 complex. This stabilization enhances the ubiquitination and subsequent proteasomal degradation of Nrf2, the master regulator of antioxidant response, thereby crippling cell’s ability to neutralize lipid peroxides. In summary, this study unveils a novel virulence mechanism wherein SS2 effector Stk1 promotes Nrf2 degradation to trigger ferroptosis, ultimately leading to the disruption of respiratory epithelial barrier. These findings suggest that inhibiting ferroptosis could represent a promising therapeutic strategy for clinical prevention and treatment of SS2 infections.

## Introduction

*Streptococcus suis* (SS) is a Gram-positive, opportunistic zoonotic pathogen that poses a significant global public health threat [1]. Though it typically colonizes the upper respiratory tract of pigs asymptomatically, it can cause severe invasive disease. Based on its capsular polysaccharide antigens, S. suis is classified into 33 serotypes, with multiple serotypes often coexisting in the same host, creating a reservoir for both porcine and human infections [2,3]. Of these, serotype 2 (SS2) is the most frequently isolated clinical strain and is recognized as one of the most virulent in both swine and humans [4]. In pigs, SS2 invades primarily through the respiratory tract, leading to pneumonia, septicaemia, and arthritis, which results in substantial economic losses in the swine industry [5]. In humans, infection typically occurs through occupational exposure to infected pigs or contaminated pork products and can manifest as life-threatening meningitis and streptococcal toxic shock-like syndrome (STSLS) [6]. While respiratory pathogens have evolved diverse strategies to breach host epithelial barriers to establish systemic infection [7], the precise molecular mechanisms underpinning the trans-epithelial invasion of SS2 remain poorly understood.

The respiratory epithelial barrier serves as the primary line of defense against inhaled pathogens and environmental insults, performing critical functions in physical protection, mucociliary clearance, and immune surveillance [8]. The integrity of this barrier is predominantly maintained by intercellular junctional complexes, which seal the paracellular space to prevent pathogen penetration [9,10]. These complexes are mainly composed of tight junctions (TJs), formed by proteins such as Claudins and Ocludin, and adherens junctions (AJs), which are primarily mediated by E-cadherin. These transmembrane proteins link adjacent cells and anchor to the intracellular actin cytoskeleton via adaptor proteins (e.g. ZO-1, catenins), thereby regulating epithelial polarity and mechanical stability [11,12]. Importantly, the expression of these core junctional proteins can be suppressed by transcriptional repressors, leading to increased paracellular permeability and a compromised barrier – a vulnerability frequently exploited by invading pathogens [10,13].

Ferroptosis is a distinct form of regulated cell death characterized by iron-dependent accumulation of lipid peroxides [14]. This process is initiated when excessive intracellular iron catalyses the production of reactive oxygen species (ROS), which drive the peroxidation of polyunsaturated fatty acids (PUFA) within cell membranes, leading to membrane damage and cell [15]. Cellular defense against ferroptosis is primarily orchestrated by the antioxidant system. The system xc^−^ imports cystine for the synthesis of glutathione (GSH), which is then utilized by glutathione peroxidase 4 (Gpx4) to detoxify lipid peroxides by reducing them to non-toxic lipid alcohols [16,17]. The transcription factor Nrf2 is the master regulator of this antioxidant response, activating the expression of cytoprotective genes, including Gpx4 and Slc7a11, to suppress ferroptosis [18]. Initially described in the context of cancer, neurodegenerative diseases, and ischemia-reperfusion injury [19]. Ferroptosis is now emerging as a critical process in host–pathogen interactions [20]. Several pathogens, including respiratory bacteria such as *Mycobacterium tuberculosis* and *Pseudomonas aeruginosa*, have been shown to manipulate host ferroptosis to facilitate their replication and dissemination [21,22]. However, the potential interplay between S. suis and ferroptosis, and whether this cell death pathway contributes to SS2-mediated barrier disruption, has not yet been investigated.

In this study, we investigated the hypothesis that SS2 exploits ferroptosis to breach the respiratory epithelial barrier. We demonstrate that SS2 infection sensitizes epithelial cells to ferroptosis by disabling the central Nrf2-mediated antioxidant pathway. We identify the bacterial kinase Stk1 as the key virulence factor that orchestrates this process by promoting the ubiquitin-dependent degradation of Nrf2. The resulting collapse of the antioxidant defense system leads to overwhelming lipid peroxidation, the dismantling of cell–cell junctions, and ultimately, the disruption of epithelial barrier integrity. Our work thus reveals a novel virulence strategy employed by SS2 to facilitate its dissemination from the respiratory tract to systemic sites and identifies the host ferroptosis pathway as a potential therapeutic target for mitigating S. suis infection.

## Results

### SS2 infection disrupts the respiratory epithelial barrier in vivo and in vitro

**Figure 1.**
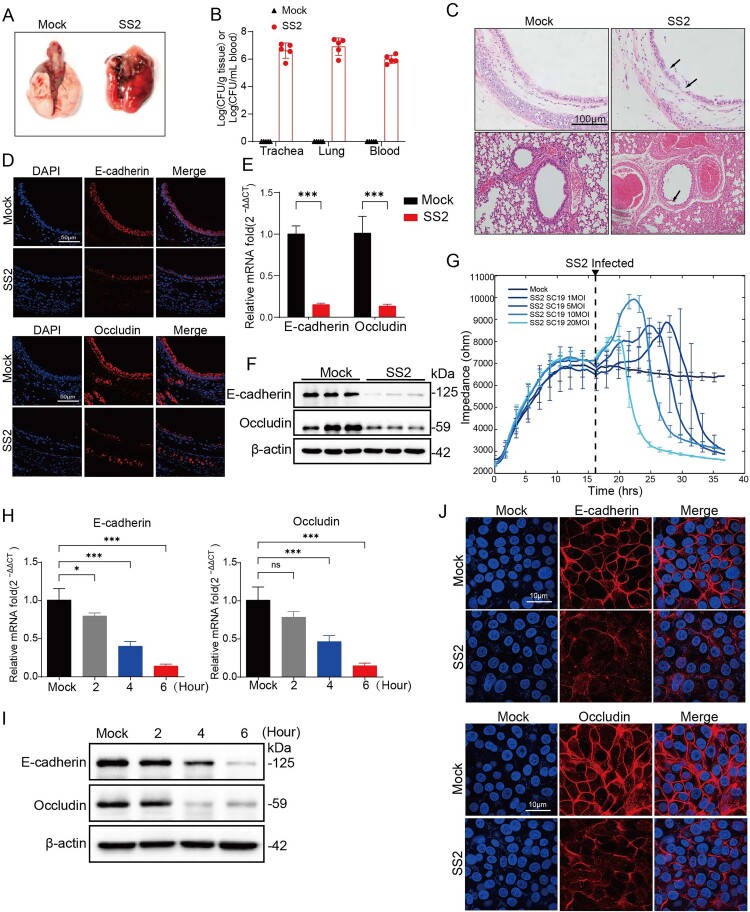
*S. suis* infection disrupts the respiratory epithelial barrier in vivo and in vitro. (A-F) Mice were intranasally infected with SS2 (6 × 10^8^ CFU) or PBS as a control for 18 h. (A) Representative images showing gross pathological lesions in the trachea and lungs. (B) Bacterial loads in the trachea, lungs, and blood were quantified by plating serial dilutions. (C) Representative H&E-stained sections of tracheal and lung tissues. Scale bars, 100 µm. (D) Immunofluorescence analysis of E-cadherin and Occludin localization in the tracheal epithelium. Nuclei were counterstained with DAPI (blue). Scale bar, 50 µm. (E) Relative mRNA levels of E-cadherin and Occludin in tracheal tissues were determined by qRT-PCR. (F) Western blot analysis of E-cadherin and Occludin protein levels in tracheal tissue lysates. (G-J) NPTr cells were infected with SS2 to assess in vitro barrier dysfunction. (G) Real-time monitoring of transepithelial electrical resistance (TEER) of NPTr cell monolayers following infection with SS2 at the indicated multiplicity of infection (MOI), measured by electric cell-substrate impedance sensing (ECIS). (H) Time-course analysis of E-cadherin and Occludin mRNA levels by qRT-PCR in NPTr cells infected with SS2 (MOI = 10). (I) Time-course Western blot analysis of E-cadherin and Occludin protein levels post-infection. (J) Immunofluorescence analysis of E-cadherin and Occludin at 6 h post-infection (hpi). Scale bar, 10 µm. All quantitative data are represented as mean ± SD from triplicate independent experiments. Statistical significance was determined by one-way ANOVA test (*p < 0.05; **p < 0.01; ***p < 0.001, ns, no significant).

To establish that *S. suis* serotype 2 (SS2) compromises the respiratory barrier during infection, we first utilized a murine intranasal challenge model. Infection with SS2 resulted in severe tracheal and pulmonary lesions (Figure 1(A)) and led to significantly higher bacterial burdens in the trachea, lungs, and blood compared to the control group (Figure 1(B)). Histopathological analysis confirmed substantial barrier damage, including ciliary fragmentation and denudation of the tracheal epithelium, alongside alveolar collapse and inflammatory cell infiltration in the lungs (Figure 1(C)). This structural damage was accompanied by a significant reduction in the expression of the junctional proteins E-cadherin and Occludin at both the protein and mRNA levels in the murine tracheal epithelium, as confirmed by immunofluorescence, Western blot, and qPCR (Figure 1(D-F)). These findings, indicative of increased paracellular permeability, were consistent with observations in an SS2-infected piglet model (Supplementary Figure 1A-F), demonstrating the cross-species relevance of this pathogenic mechanism.

To further investigate this phenomenon at the cellular level, we employed an in vitro model using polarized porcine respiratory epithelial cells (NPTr). SS2 infection induced a multiplicity of infection (MOI)-dependent decrease in barrier function, as measured by a decline in transepithelial electrical resistance (TEER) using electric cell–substrate impedance sensing (ECIS) (Figure 1(G)). Time-course analyses further revealed a progressive loss of E-cadherin and Occludin at both the transcriptional and translational levels (Figure 1(H–J)). Collectively, these findings from multiple models demonstrate that SS2 infection compromises the integrity of the respiratory epithelial barrier by dismantling key intercellular junctional proteins, thereby facilitating bacterial invasion and dissemination.

### SS2 infection induces ferroptosis in respiratory epithelial cells

**Figure 2.**
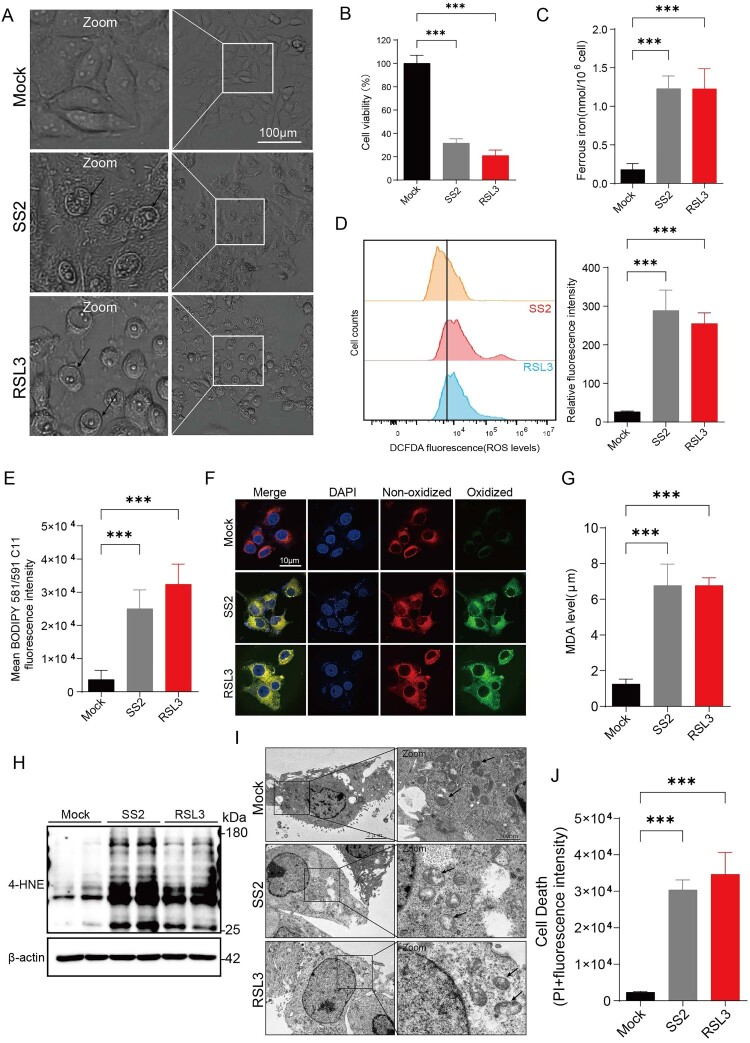
*S. suis* induces hallmark features of ferroptosis in respiratory epithelial cells. (A-J) NPTr cells were infected with SS2 (MOI = 10) or treated with the ferroptosis inducer RSL3 (1 µM) for 6 h. (A) Representative phase-contrast microscopy images showing cellular morphology. Scale bar, 100 µm. (B) Cell viability was assessed by the CCK-8 assay. (C) Intracellular labile ferrous iron (Fe^2+^) levels were quantified using a colorimetric assay kit. (D) Reactive oxygen species (ROS) production was measured by flow cytometry analysis of H2DCFDA fluorescence. MFI, mean fluorescence intensity. (E) Lipid peroxidation was visualized by confocal microscopy using the C11-BODIPY 581/591 probe. Scale bar, 10 µm. (F) Quantification of lipid peroxidation by measuring C11-BODIPY fluorescence using a plate reader. (G) Malondialdehyde (MDA) levels were quantified using a thiobarbituric acid reactive substances (TBARS) assay. (H) Western blot analysis of 4-hydroxynonenal (4-HNE) protein adducts. (I) Mitochondrial ultrastructure was analysed by transmission electron microscopy. Arrows indicate shrunken mitochondria with loss of cristae. Scale bars, 2 µm (main image) and 500 nm (inset). (J) Cell death was quantified by measuring propidium iodide (PI) fluorescence. All quantitative data are represented as mean ± SD from triplicate independent experiments. Statistical significance was determined by one-way ANOVA test (*p < 0.05; **p < 0.01; ***p < 0.001, ns, no significant).

Having established that SS2 disrupts the epithelial barrier, we next investigated the underlying mechanism of cell death, hypothesizing a role for ferroptosis – a pathway not previously linked to *S. suis* pathogenesis. We found that SS2 infection of NPTr cells induced morphological changes characteristic of ferroptosis, including intact nuclear membranes without evidence of blebbing, similar to cells treated with the classical ferroptosis inducer RSL3 (Figure 2(A)) [23]. SS2 infection induced significant cytotoxicity (Figure 2(B)) and a corresponding increase in the intracellular Fe²^+^ levels (Figure 2(C)). A key driver of ferroptosis is iron-dependent lipid peroxidation. Accordingly, SS2-challenged cells displayed a significant accumulation of reactive oxygen species (ROS), as measured by flow cytometry using H2DCFDA probe (Figure 2(D)), and pronounced lipid peroxidation, as demonstrated by both fluorometry and confocal microscopy using the C11-BODIPY 581/591 probe (Figure 2(E, F)). Furthermore, SS2 infection led to a marked increase in the classical ferroptosis biomarkers malondialdehyde (MDA) and 4-hydroxynonenal (4-HNE) to levels comparable to RSL3 treatment (Figure 2(G, H)). Ultrastructural analysis by transmission electron microscopy revealed mitochondrial vacuolization and the disappearance of cristae, which are hallmark features of ferroptosis (Figure 2(I, J)). Collectively, these data provide comprehensive evidence that SS2 infection is a potent inducer of ferroptosis in respiratory epithelial cells.

### Pharmacological inhibition of ferroptosis protects against SS2-Induced epithelial barrier disruption

**Figure 3.**
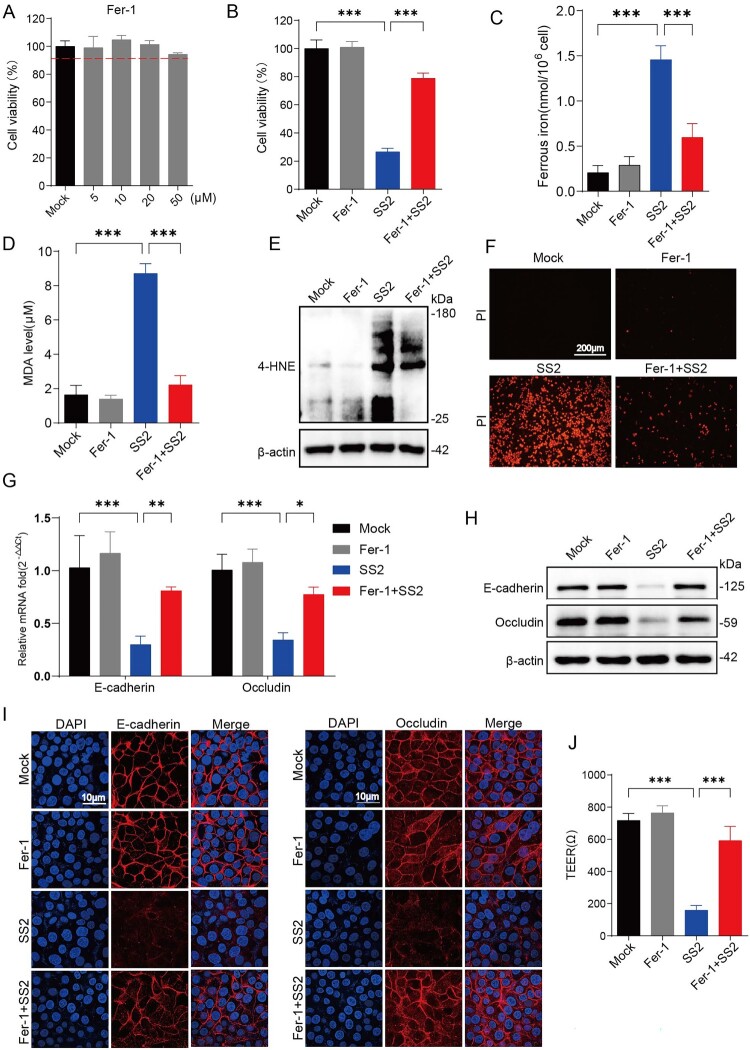
Pharmacological inhibition of ferroptosis protects against SS2-induced epithelial barrier disruption. (A) Viability of NPTr cells treated with the indicated concentrations of Ferrostatin-1 (Fer-1) for 6 h, assessed by the CCK-8 assay. (B-J) NPTr cells were pretreated with Fer-1 (20 µM) or vehicle (DMSO) for 2 h prior to infection with SS2 (MOI = 10) for 6 h. (B) Cell viability was determined by the CCK-8 assay. (C) Intracellular Fe^2+^ levels were measured using ferrous ion detection assay kit. (D) MDA concentration in the cells was measured to assess lipid peroxidation levels. (E) Western blot analysis of 4-HNE protein adducts. (F) Representative fluorescence microscopy images of cells stained with PI to visualize dead cells. Scale bar, 200 µm. (G) qRT-PCR analysis of E-cadherin and Occludin mRNA levels. (H) Western blot analysis of E-cadherin and Occludin protein levels. (I) Immunofluorescence analysis of E-cadherin and Occludin using confocal microscopy. Scale bar, 10 µm. (J) TEER measurements of NPTr monolayers grown on Transwell inserts. All quantitative data are represented as mean ± SD from triplicate independent experiments. Statistical significance was determined by one-way ANOVA test (*p < 0.05; **p < 0.01; ***p < 0.001, ns, no significant).

To confirm the causal role of ferroptosis in SS2-mediated cytotoxicity and barrier dysfunction, we utilized the specific ferroptosis inhibitor ferrostatin-1 (Fer-1) [24]. While Fer-1 treatment alone had no effect on cell viability, its co-treatment with SS2 effectively rescued infected NPTr cells from cell death (Figure 3(A, B)). This protective effect was associated with a significant reduction in intracellular Fe²^+^ levels (Figure 3C) and a marked suppression of the lipid peroxidation markers MDA and 4-HNE (Figure 3(D, E)). Consistent with these findings, Fer-1 treatment preserved plasma membrane integrity, as indicated by a decrease in propidium iodide (PI) uptake (Figure 3(F)).

We next sought to determine whether inhibiting ferroptosis could also rescue the SS2-induced loss of epithelial barrier integrity. Indeed, Fer-1 treatment significantly mitigated the SS2-induced downregulation of E-cadherin and Occludin at both the mRNA and protein levels (Figure 3(G–I)). Functionally, Fer-1 co-treatment substantially attenuated the drop in TEER caused by SS2 infection, demonstrating preservation of barrier function (Figure 3(J)). Moreover, pharmacological inhibition of ferroptosis notably constrained the proliferation ability of SS2 (Supplementary Figure 2A, B). These findings establish a direct link between SS2-induced ferroptosis and the disruption of the respiratory epithelial barrier.

### Lipid peroxidation products mediate junctional disruption via snail1 upregulation

**Figure 4.**
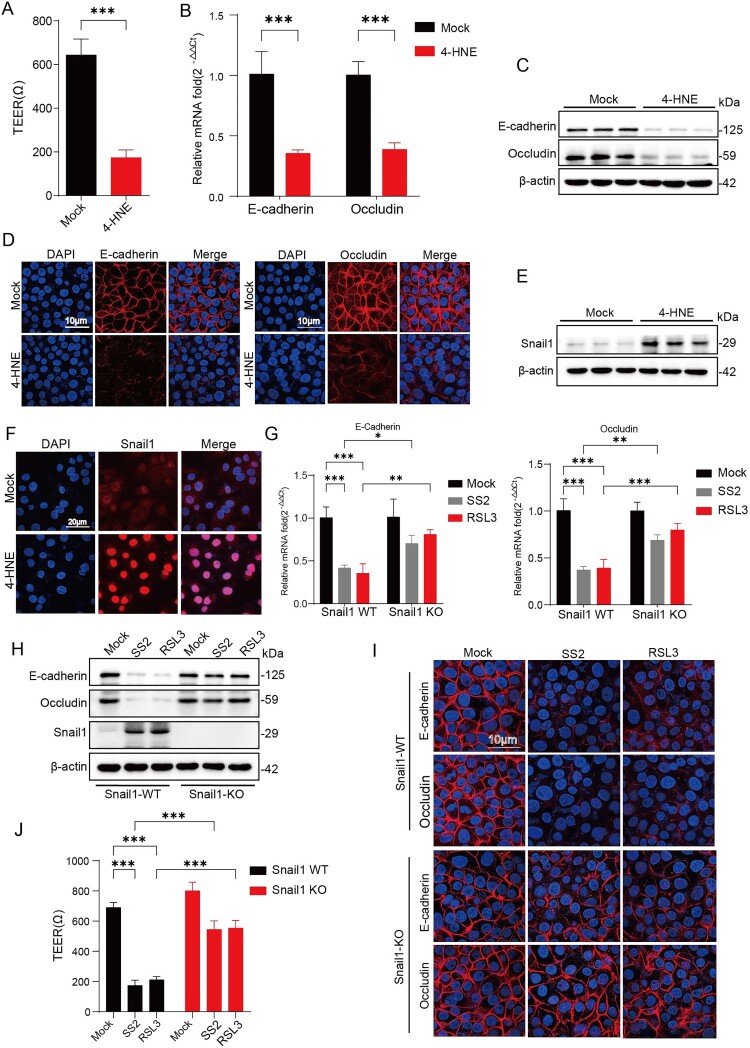
Lipid peroxidation products dismantle epithelial junctions via Snail1 upregulation. (A) The Transwell model was used to assess epithelial barrier permeability. NPTr cells were treated with 4-HNE (20 μM) or vehicle (DMSO) for 6 h, and TEER values were measured using a resistance meter. (B-F) NPTr cells were treated with 4-HNE (20 μM) or vehicle (DMSO) for 6 h.(B) Cell genomic DNA was prepared and extracted, and qPCR was performed to analyse the mRNA levels of E-cadherin and Occludin. (C) Western blot analysis was used to measure the protein levels of E-cadherin and Occludin in cell lysates. (D) Expression levels of E-cadherin and Occludin in cells were analysed using confocal microscopy (scale bar = 10 μm). (E) Snail-1 protein levels in the lysates were detected by Western blot. (F) Snail-1 expression and localization in cells were analysed using confocal microscopy (scale bar = 20 μm).(G-I) Snail-1-WT or Snail-1-KO cells were infected with SS2 (MOI = 10) and treated with RSL3 (1 μM) for 6 h.(G) Cell genomic DNA was prepared and extracted, and qPCR was performed to analyse the mRNA levels of E-cadherin and Occludin.(H) Western blot analysis was used to measure the protein levels of E-cadherin and Occludin in cell lysates.(I) Expression levels of E-cadherin and Occludin in cells were analysed using confocal microscopy (scale bar = 10 μm).(J) The Transwell model was used to assess epithelial barrier permeability. Snail-1-WT or Snail-1-KO cells were infected with SS2 (MOI = 10) and treated with RSL3 (1 μM) for 6 h. TEER values were measured using a resistance meter. All quantitative data are represented as mean ± SD from triplicate independent experiments. Statistical significance was determined by one-way ANOVA test (*p < 0.05; **p < 0.01; ***p < 0.001, ns, no significant).

During ferroptosis, lipid peroxidation converts membrane lipids into a range of reactive aldehyde byproducts, among which 4-HNE and MDA are the most extensively studied. These compounds contribute to and amplify the downstream biological consequences associated with ferroptotic cell death [25]. Given that ferroptosis inhibition rescued barrier function, we hypothesized that the accumulation of lipid peroxides, the hallmark of ferroptosis, was responsible for the degradation of junctional proteins. To test this, we treated NPTr cells directly with 4-HNE, a major product of lipid peroxidation. This treatment alone was sufficient to cause a significant reduction in TEER (Figure 4(A)) and a corresponding decrease in E-cadherin and Occludin mRNA levels (Figure 4(B)). The reduction in these junctional proteins was confirmed at the protein level by Western blot and immunofluorescence assays (Figure 4(C, D)), indicating that lipid peroxidation products can directly compromise barrier integrity by downregulating junctional protein expression.

The zinc-finger transcription factor Snail1 is a known repressor of epithelial junction proteins [26]. We observed that 4-HNE treatment led to a significant upregulation and nuclear localization of Snail1 in NPTr cells, as demonstrated by WB analysis and Laser confocal microscopy (Figure 4(E, F)). To directly test the role of Snail1 in this process, we generated a Snail1 knockout (KO) NPTr cell line. Critically, Snail1-KO cells were significantly protected from the SS2-induced downregulation of E-cadherin and Occludin at both the mRNA and protein levels compared to wild-type (WT) cells (Figure 4(G–I)). This protection translated to a significant preservation of barrier function, as evidenced by higher TEER values in infected Snail1-KO cells (Figure 4(J)). Collectively, these results outline a mechanism through which SS2-induced lipid peroxidation products, such as 4-HNE, upregulate the transcriptional repressor Snail1. Subsequently, Snail1 regulates the down-regulation of junction protein expression, leading to the dismantling of the epithelial barrier.

### SS2 triggers ferroptosis by promoting the ubiquitin-dependent degradation of Nrf2

**Figure 5.**
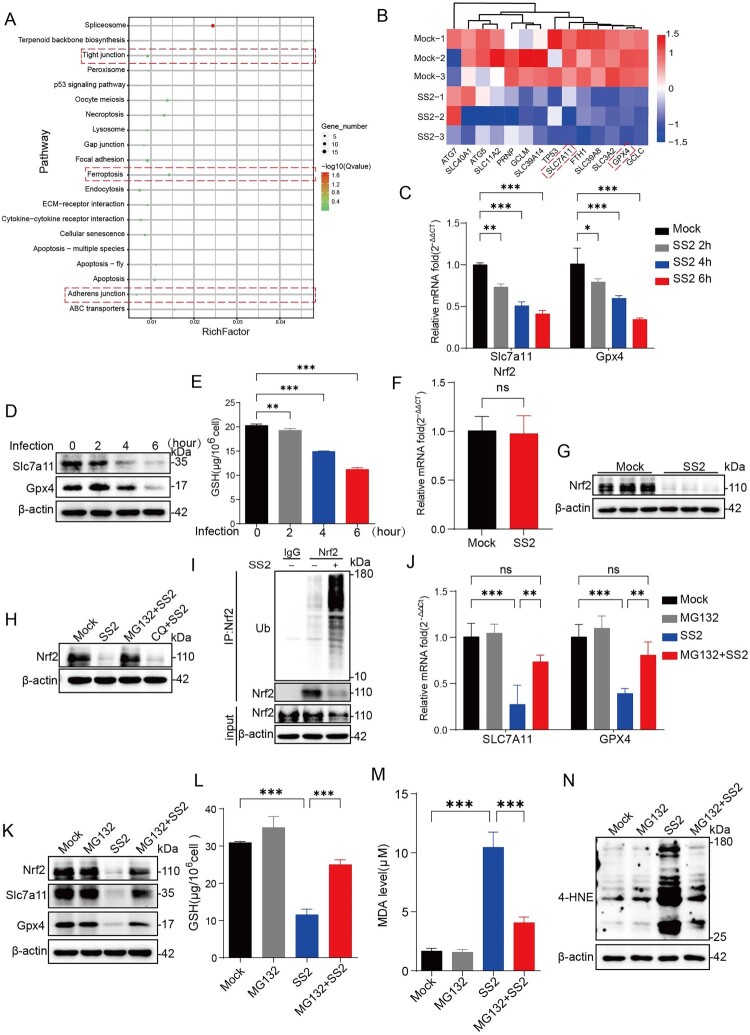
SS2 promotes ferroptosis by inducing the ubiquitin-dependent degradation of Nrf2. (A-B) NPTr cells were infected with SS2 (MOI = 10) for 6 h, and transcriptomic analysis was performed by RNA-seq. (A) KEGG pathway enrichment analysis of differentially expressed genes (DEGs). (B) Heatmap showing the expression of key antioxidant and ferroptosis-related genes. Red and blue indicate up – and down-regulated genes, respectively. (C-E) Time-course analysis of NPTr cells infected with SS2 (MOI = 10). (C) qRT-PCR analysis of Slc7a11 and Gpx4 mRNA levels. (D) Western blot analysis of Slc7a11 and Gpx4 protein levels. (E) Intracellular GSH levels measured using a GSH assay kit at different time points (2, 4, and 6 hpi). (F-I) NPTr cells were infected with SS2 (MOI = 10) for 6 h. (F) qRT-PCR analysis of Nrf2 mRNA levels. (G) Western blot analysis of Nrf2 protein levels. (H) Nrf2 protein levels in cells pretreated with the proteasome inhibitor MG132 (5 µM) or the lysosome inhibitor chloroquine (CQ, 25 µM) prior to infection. (I) Co-IP analysis of Nrf2 ubiquitination. Cell lysates were immunoprecipitated (IP) with an anti-Nrf2 antibody and immunoblotted (IB) with an anti-ubiquitin antibody. (J-N) NPTr cells were pretreated with MG132 (5 µM) for 2 h before SS2 infection (MOI = 10) for 6 h. (J) qRT-PCR analysis of Slc7a11 and Gpx4 mRNA. (K) Western blot analysis of Nrf2, Slc7a11, and Gpx4 proteins. (L) Intracellular GSH levels were measured by using a GSH assay kit. (M) MDA levels were measured with an MDA assay kit to evaluate the lipid peroxidation. (N) Western blot analysis of 4-HNE adducts. All quantitative data are represented as mean ± SD from triplicate independent experiments. Statistical significance was determined by one-way ANOVA test (*p < 0.05; **p < 0.01; ***p < 0.001, ns, no significant).

To elucidate the molecular mechanism by which SS2 induces ferroptosis, we performed RNA sequencing (RNA-seq) on SS2-infected NPTr cells. The comparison of FPKM density distributions for genes and transcripts (Supplementary Figure 3A) and the principal component analysis (PCA) of high-throughput transcriptome sequencing samples (Supplementary Figure 3B) collectively satisfied the quality control criteria for RNA-seq data. Clustering analysis and differential gene expression volcano plots identified 196 significantly differentially expressed genes (Supplementary Figure 3C, D), with KEGG pathway analysis showing strong enrichment for pathways related to adherens junctions, tight junctions, and ferroptosis (Figure 5(A)). Specifically within the ferroptosis pathway, we observed a significant downregulation of key host antioxidant genes, including *Gpx4, Slc7a11, Gclc, and Gclm* (Figure 5(B)). We validated these findings, confirming that SS2 infection caused a time-dependent decrease in the mRNA and protein levels of Gpx4 and Slc7a11, as well as a reduction in cellular GSH levels (Figure 5(C–E)).

Nrf2 binds to the antioxidant response elements (AREs) in the promoter regions of target genes, including Gpx4, Slc7a11, and genes involved in NADPH metabolism, thereby playing a crucial role in the cellular antioxidant defense system. The down-regulation of these key antioxidant genes motivated us to conduct an investigation into their primary transcriptional regulator, Nrf2. Additionally, Nrf2 enhances GSH synthesis by upregulating the expression of rate-limiting enzymes in the GSH biosynthetic pathway [27,28]. Interestingly, while Nrf2 mRNA levels were unchanged following SS2 infection (Figure 5(F)), Nrf2 protein levels were significantly reduced (Figure 5(G)), suggesting post-transcriptional regulation. To discern the degradation pathway, we treated infected cells with inhibitors of the proteasome (MG132) or lysosome (chloroquine). MG132, but not chloroquine, restored Nrf2 protein levels, implicating the ubiquitin-proteasome system in its degradation (Figure 5(H)). Co-immunoprecipitation assays subsequently confirmed that SS2 infection markedly increased the ubiquitination of Nrf2 (Figure 5(I)).

To functionally link Nrf2 degradation to the ferroptotic phenotype, we showed that blocking proteasomal degradation with MG132 not only stabilized Nrf2 but also restored the expression of its downstream targets Gpx4 and Slc7a11 (Figure 5(I–K)). This restoration of the antioxidant response led to an increase in GSH levels and a significant inhibition of lipid peroxidation in SS2-infected cells (Figure 5(L–N)). Together, these data demonstrate that SS2 dismantles the cellular antioxidant defense system by promoting the ubiquitin-proteasome-dependent degradation of Nrf2, thereby sensitizing epithelial cells to ferroptosis.

### SS2 effector Stk1 Hijacks the host Keap1-Nrf2 axis to induce Nrf2 degradation

To identify the SS2 virulence factor responsible for inducing ferroptosis, we screened 11 major virulence factors for their ability to sensitize NPTr cells to RSL3-induced lipid peroxidation, an established marker of ferroptosis [29]. Overexpression of the eukaryotic-like serine/threonine kinase 1 (Stk1) and the suilysin (Sly) protein resulted in the highest levels of lipid ROS and cell death (Supplementary Figure 4A, B). Based on previous reports implicating bacterial serine/threonine kinases in host ubiquitination pathways, we focused on Stk1 as the primary candidate for mediating Nrf2 degradation.

**Figure 6.**
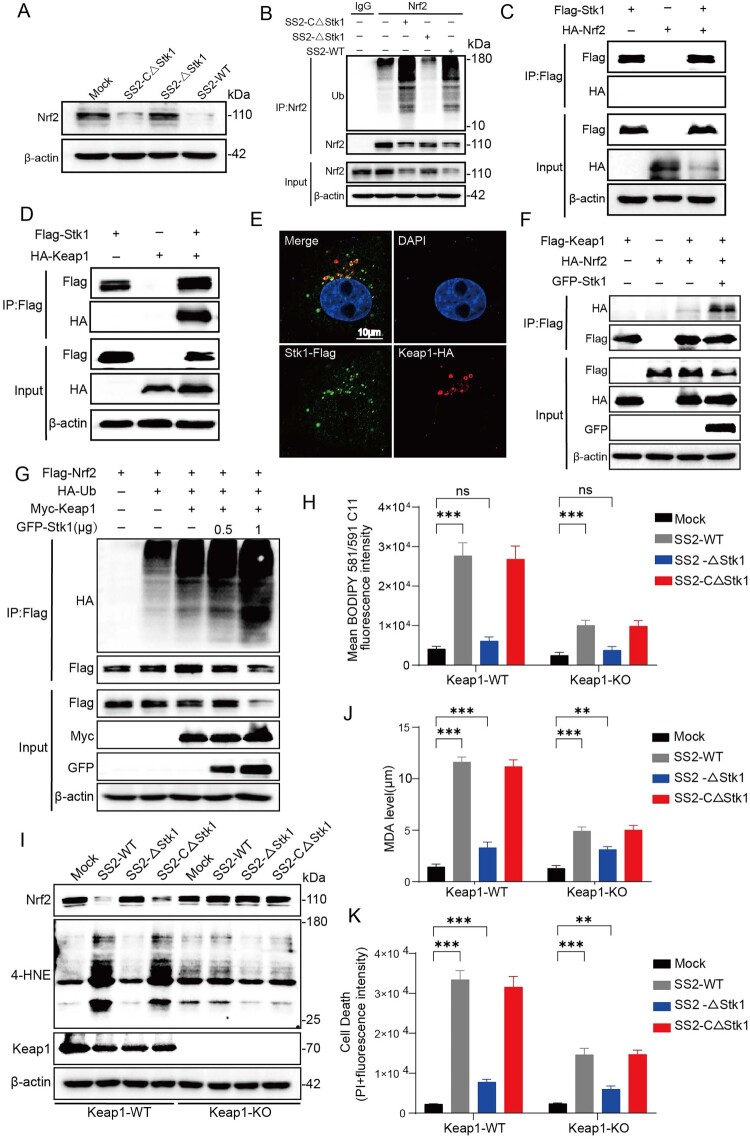
The SS2 effector Stk1 hijacks the Keap1-Nrf2 axis to promote Nrf2 degradation. (A-B) NPTr cells were infected with SS2 wild-type (WT), the ΔStk1 mutant, or the complemented strain (CΔStk1) (MOI = 10). (A) Western blot analysis of Nrf2 protein levels at 6 hpi. (B) Co-IP analysis of Nrf2 ubiquitination at 6 hpi. (C-G) Co-IP experiments were performed in HEK293 T cells transfected with the indicated plasmids for 24 h. (C) Analysis of the interaction between Stk1-FLAG and Nrf2-HA. (D) Analysis of the interaction between Stk1-FLAG and Keap1-HA. (E) Immunofluorescence analysis showing cytoplasmic co-localization of Stk1-FLAG (green) and Keap1-HA (red). Scale bar, 10 µm. (F) Co-IP showing that Stk1-GFP enhances the interaction between Keap1-FLAG and Nrf2-HA. (G) Co-IP showing that Stk1-GFP enhances the Keap1-myc-mediated ubiquitination of Nrf2-FLAG. (H-K) Keap1 WT or KO NPTr cells were infected with SS2 strains (MOI = 10) for 6 h. (H) Lipid peroxidation quantified by C11-BODIPY fluorescence. (I) Western blot analysis of Nrf2 and 4-HNE levels. (J) MDA concentration was measured to evaluate lipid peroxidation levels. (K) Cell death quantified by PI fluorescence. Data are presented as mean ± SD from three independent experiments. All quantitative data are represented as mean ± SD from triplicate independent experiments. Statistical significance was determined by one-way ANOVA test (*p < 0.05; **p < 0.01; ***p < 0.001, ns, no significant).

The SS2 *Stk1* gene deletion mutant ΔStk1 and its complemented strain CΔStk1 were constructed to investigate the role of Stk1 in Nrf2 ubiquitination-mediated degradation during SS2 infection of NPTr cells. Experiments using the SS2 WT, ΔStk1, and CΔStk1 strains confirmed that Stk1 is essential for the SS2-induced degradation of Nrf2 protein (Figure 6(A)) and the subsequent increase in Nrf2 ubiquitination (Figure 6(B)). Since immunoprecipitation assays revealed no direct interaction between Stk1 and Nrf2 (Figure 6(C)), we hypothesized that Stk1 acts via an intermediary. We turned our attention to Keap1 (Kelch-like ECH-associated protein 1), the substrate adaptor for the Cul3-E3 ubiquitin ligase complex that targets Nrf2 for degradation [30]. The Keap1-Nrf2 axis is also involved in the regulation of ferroptosis [31]. Co-immunoprecipitation and immunofluorescence assays demonstrated that Stk1 directly interacts with and co-localizes with Keap1 in the cytoplasm (Figure 6(D,E)). Critically, the co-expression of Stk1 enhanced the interaction between Keap1 and Nrf2 (Figure 6(F)) and promoted the ubiquitination of Nrf2 (Figure 6(G)). These findings collectively suggest that SS2 infection promotes the interaction between Stk1 and Keap1, thereby stabilizing the Keap1-Nrf2 complex. This stabilization fosters Nrf2 ubiquitination and subsequent degradation.

To definitively establish the role of the Keap1-Nrf2 axis in this process, we generated a Keap1 knockout (KO) NPTr cell line using the CRISPR/Cas9 system. In Keap1-KO cells, SS2 infection alleviated induce Nrf2 degradation, regardless of the presence of Stk1 (Figure 6(H)). Consequently, Keap1-KO cells were protected from SS2-induced ferroptosis, exhibiting significantly lower levels of lipid ROS, MDA, and 4-HNE, and reduced cell death compared to Keap1-WT cells (Figure 6(I,K)). These findings indicate that the SS2 effector Stk1 hijacks the host Keap1-Nrf2 axis, interacting with Keap1 to stabilize its association with Nrf2, thereby promoting Nrf2 ubiquitination and degradation to trigger epithelial ferroptosis.

### Stk1-Mediated ferroptosis drives respiratory barrier disruption and bacterial dissemination in vivo

**Figure 7.**
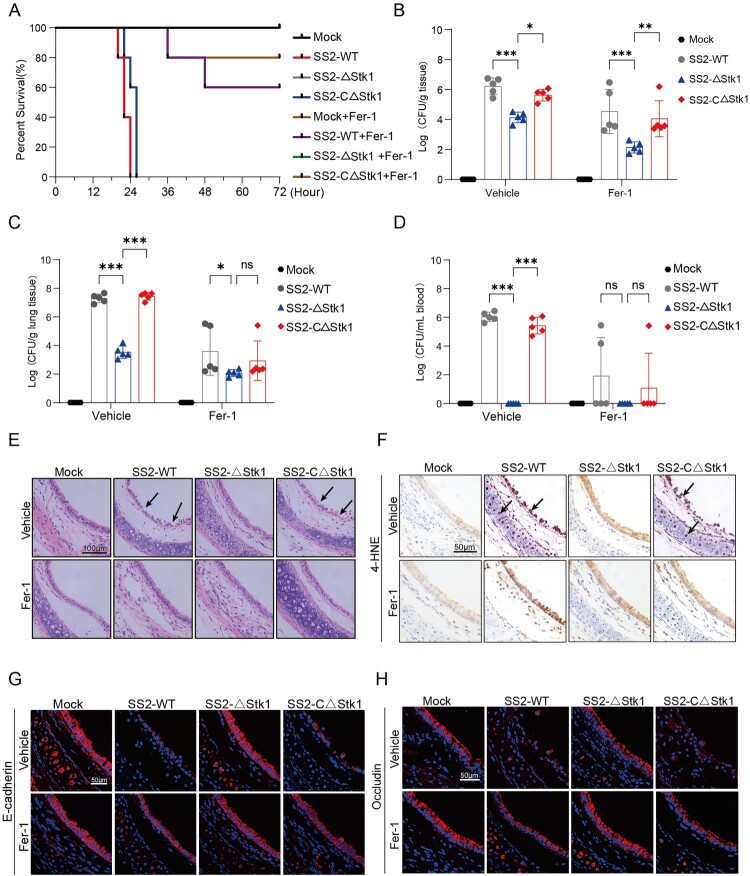
Stk1-mediated ferroptosis drives respiratory barrier disruption and bacterial dissemination in vivo. (A-H) Mice were intratracheally infected with SS2 WT, ΔStk1, or CΔStk1 (6 × 10^8^ CFU) and treated with Fer-1 or vehicle via tail vein injection. (A) The Kaplan-Meier survival curve of infected mice (n = 10) was continuously monitored. (B-D) Bacterial loads in the (B) trachea, (C) lungs, and (D) blood at 18 hpi. (E) Representative H&E-stained images of tracheal tissues. Scale bar, 100 µm. (F) Immunohistochemistry analysis of 4-HNE adducts in tracheal tissues. Scale bar, 50 µm. (G-H) Immunofluorescence analysis of E-cadherin and Occludin in mouse tracheal tissues by confocal microscopy. Scale bars, 50 µm. Data are presented as mean ± SD (n = 10 mice per group for survival; n = 5 for other panels). Statistical significance was determined by the log-rank (Mantel-Cox) test for survival curves and one-way ANOVA test for other panels (*p < 0.05; **p < 0.01; ***p < 0.001, ns, no significant).

Finally, to validate the physiological relevance of the Stk1-ferroptosis axis in vivo, we utilized our murine infection model. Consistent with our in vitro data, mice infected with the WT SS2 or the complemented strain (SS2 CΔStk1) exhibited more severe pathological damage to the tracheal epithelium and lungs, and supported higher bacterial loads, compared to mice infected with the ΔStk1 mutant (Figure 7(A–E)). At the molecular level, tracheal tissues from WT – and CΔStk1-infected mice showed elevated 4-HNE staining, markedly reduced Nrf2 protein levels (despite unchanged mRNA levels), and significant downregulation of the Nrf2 targets Slc7a11 and Gpx4 (Figure 7(F) and Supplementary Figure 5A-E).

Crucially, treatment with the ferroptosis inhibitor Fer-1 largely reversed these effects in mice infected with WT SS2, restoring Nrf2 protein levels and antioxidant gene expression while reducing lipid peroxidation (Figure 7(F) and Supplementary Figure 5A-E). This intervention also protected barrier integrity, as evidenced by the preserved expression of E-cadherin and Occludin (Figure 7(G, H) and Supplementary Figure 5F-I). These in vivo results confirm that Stk1 is a key virulence factor that triggers ferroptosis to dismantle the respiratory epithelial barrier, thereby promoting bacterial invasion and systemic dissemination.

## Discussion

*Streptococcus suis (S. suis)* is a formidable zoonotic pathogen, with serotype 2 (SS2) representing the most prevalent and virulent clinical isolate, posing a significant threat to both swine health and public safety [32]. The initial and critical step in the pathogenesis of many invasive diseases is the breach of the host's mucosal barriers, such as the respiratory epithelium. While pathogens have evolved diverse strategies to overcome this defense, the precise molecular mechanisms underpinning this process remain incompletely understood [33]. Host-regulated cell death pathways are often co-opted by pathogens to facilitate their invasion and proliferation [34]. Ferroptosis, a recently identified form of iron-dependent cell death, has been extensively studied in non-infectious diseases, including cancer, neurodegenerative diseases, and organ damage [35]. Its role in bacterial pathogenesis is an emerging field, and evidence indicates that pathogens may exploit ferroptosis to increase their pathogenicity during an infection [20]. Here, we provide the first evidence that SS2 infection triggers ferroptosis in respiratory epithelial cells, dismantling the host antioxidant system to disrupt the epithelial barrier. Although SS2 activates multiple cellular stress pathways and induces various forms of cellular injury, our results establish ferroptosis as the core mechanism underlying epithelial barrier dysfunction. Importantly, we demonstrate that pharmacological inhibition of ferroptosis not only preserves barrier integrity but also restricts SS2 proliferation, highlighting this pathway as a promising therapeutic target for combating SS2 infections.

The structural integrity of the respiratory epithelium is maintained by AJs and TJs, dynamic structures that are frequently targeted by microbial virulence factors [36,37]. Several studies have demonstrated that pathogens can enhance paracellular permeability by disrupting the localization of AJs and TJs in epithelial cells, thereby promoting their translocation across the epithelial barrier. Our findings indicate that SS2 infection downregulates the key junctional proteins, including E-cadherin and Occludin, which is consistent with established mechanisms of bacterial pathogenesis. For instance, *P. aeruginosa* utilizes the type III secretion system effector ExoS to disrupt epithelial junctions, leading to increased permeability of the respiratory epithelial barrier [38]. *Glaesserella parasuis* degrades TJ proteins via the autophagy-lysosome pathway to increase the permeability of the respiratory epithelial barrier [39]. Similarly, the *M. tuberculosis* serine protease Rv2569c cleaves E-cadherin to increase permeability of the alveolar epithelial barrier, thereby promoting systemic dissemination [40]. Our study adds a new dimension to this field by demonstrating that the SS2-induced loss of junctional proteins is a direct consequence of ferroptosis. This finding prompted us to investigate the mechanistic link between the execution of ferroptotic cell death and the regulation of intercellular junctions.

A central executioner of ferroptosis is the accumulation of lipid peroxidation products, with 4-HNE being a key marker of lipid peroxidation as well as a highly reactive and pathologically significant aldehyde. By forming adducts with proteins and other macromolecules, 4-HNE can profoundly alter the function of the affected molecules through a process called carbonylation [25]. The pathogenic role of 4-HNE is increasingly acknowledged in the context of infectious diseases. For instance, plasma samples from patients who died due to SARS-CoV-2 infection were found to contain higher levels of 4-HNE protein adducts compared to those from survivors, indicating a potential association between elevated 4-HNE levels and increased mortality [41]. Furthermore, influenza A virus (IAV) has been shown to induce lipid peroxidation through haemagglutinin-mediated ferroptosis. 4-HNE impairs the aggregation of mitochondrial antiviral signalling proteins (MAVS), thereby suppressing type I interferon responses and facilitating viral replication [42]. Our work reveals a novel consequence of 4-HNE accumulation: the transcriptional suppression of E-cadherin and Occludin. We discovered that the epithelial–mesenchymal transition (EMT)-related transcription factor Snail1 serves as a mediator in this process, suggesting that 4-HNE treatment upregulates Snail1, which is recognized to play a crucial role in the down-regulation of junctional protein expression. This finding is in line with studies on *Group B Streptococcus*, where the upregulation of Snail1 mediates BBB disruption by regulating the down-regulation of Occludin, ZO-1, and Claudin-5 [13]. In human corneal epithelial cells, 4-HNE has been demonstrated to induce inflammatory responses and oxidative stress, as evidenced by nuclear factor-kappa B (NF-κB) activation and ROS production [43]. We hypothesize that 4-HNE may facilitate the upregulation of Snail1 by modulating these inflammatory and oxidative stress signalling pathways. Nevertheless, whether the upregulation of Snail1 primarily results from transcriptional activation or enhanced protein stability necessitates further investigation to clarify the precise molecular mechanisms. Our results thus delineate a complete pathway from ferroptosis-induced lipid peroxidation to Snail1-mediated junctional disassembly.

To understand how SS2 triggers this cascade, we examined Nrf2, the master regulator of the cellular antioxidant response, which governs the expression of numerous antioxidant genes and plays a critical role in inhibiting ferroptosis. It is widely recognized that Nrf2 transcriptionally regulates multiple genes implicated in ferroptosis, including those involved in iron homeostasis and glutathione biosynthesis [44,45]. Under homeostatic conditions, Nrf2 is targeted for proteasomal degradation by its cytosolic repressor, Keap1. However, under oxidative conditions, oxidative stress typically disrupts this interaction, allowing Nrf2 to stabilize and translocate to the nucleus, activating a battery of protective antioxidant genes that suppress ferroptosis [46]. Our study demonstrates that SS2 undermines this protective axis. Rather than stabilizing Nrf2, SS2 infection promotes its Keap1-dependent ubiquitination and degradation. This regulatory mechanism primarily functions at the post-translational level, independent of transcriptional repression, thus disrupting the antioxidant capacity and rendering the cell more susceptible to ferroptosis. Treatment with MG132, a proteasome inhibitor, or Keap1 knockout was found to significantly alleviate SS2-induced epithelial ferroptosis and disruption of the epithelial barrier. This strategy of targeting Nrf2 is not unique to SS2. Previous studies have demonstrated that the SARS-CoV-2 ORF3a protein recruits Keap1 to facilitate Nrf2 degradation, thereby impairing cellular defense against oxidative stress and promoting ferroptosis, which may contribute to multi-organ damage in infected individuals [47]. Furthermore, *Staphylococcus aureus* infection has been shown to upregulate IFP35 expression, leading to enhanced ubiquitination and proteasomal degradation of Nrf2. This mechanism exacerbates ferroptosis and aggravates lung injury during *S. aureus* infection [28]. These convergent pathogenic strategies underscore the centrality of Nrf2 in host defense and solidify its potential as a therapeutic target. Indeed, pharmacological activation of Nrf2 has shown promise in mitigating various infectious diseases [48]. The Nrf2 agonist sulforaphane has been reported to exhibit antiviral activity against SARS-CoV-2 in both in vitro and in vivo studies [49].

The key bacterial determinant orchestrating this process was identified as Stk, a eukaryotic-like serine/threonine kinase. Such kinases are critical virulence factors in various pathogens, including *Streptococcus pneumoniae*, *M. tuberculosis*, and *P. aeruginosa*, and often play critical roles in biofilm formation, metabolism, antibiotic resistance, and virulence [50–52]. However, research regarding the role of Stks in pathogen–host interactions and their contribution to pathogenesis remains limited. For example, *S. pneumoniae* secretes StkP via exosomes, which phosphorylates Beclin 1 to induce autophagy, leading to Occludin degradation and disruption of the alveolar epithelial barrier [53]. Similarly, SS2 Stk1 promotes Claudin-5 degradation by modulating the HECTD1 ubiquitin ligase, thereby compromising the BBB [54]. Furthermore, *M. tuberculosis* PknG exhibits dual E1/E3 enzymatic activity, facilitating pathogen evasion of host innate immunity by degrading TRAF2 and TAK1 [55]. Herein, our current study uncovers a distinct and novel function for Stk1. We demonstrate that Stk1 does not function as a ubiquitin ligase itself, but rather serves as a molecular scaffold. Stk1 interacts directly with the host protein Keap1, thereby stabilizing the Keap1-Nrf2 complex and augmenting the efficiency of Nrf2 ubiquitination and subsequent degradation. This process disrupts the host’s antioxidant system, thereby inducing ferroptosis in respiratory epithelial cells. Previous studies have demonstrated that the serine/threonine kinase Stk3 promotes Keap1 phosphorylation in eukaryotes, resulting in increased ubiquitination and subsequent proteasomal degradation of Nrf2. This process suppresses the antioxidant response and contributes to the pathogenesis of septic cardiomyopathy [56]. However, the precise molecular mechanism by which Stk1 promotes the stable Keap1-Nrf2 interaction has not yet been fully elucidated. We hypothesize that Stk1 may function via either kinase-dependent or kinase-independent pathways, and that further investigation is necessary to delineate this mechanism in detail. Notably, the subversion of the host’s endogenous protein degradation machinery represents a sophisticated virulence strategy employed by pathogens.

**Figure 8.**
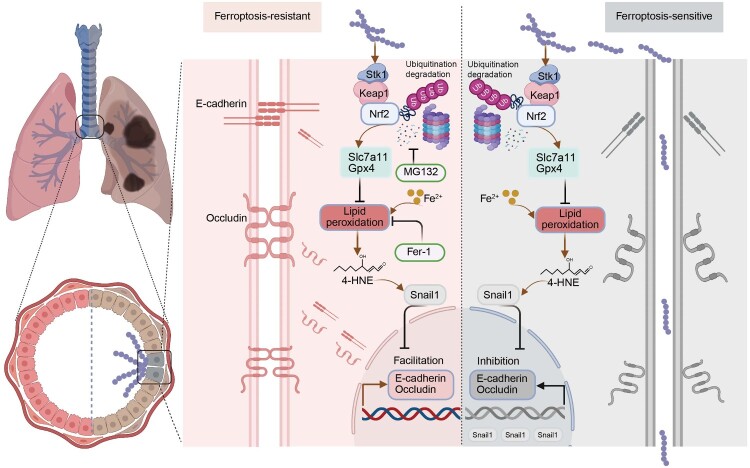
A Working Model for *S. suis* Stk1-Mediated Disruption of the Respiratory Epithelial Barrier via Ferroptosis. This model illustrates how the *S. suis* effector Stk1 interacts with host Keap1 to promote the ubiquitination and degradation of the master antioxidant regulator Nrf2. The resulting collapse of the antioxidant system sensitizes epithelial cells to ferroptosis, leading to lipid peroxidation, Snail1-mediated suppression of junctional proteins, and the ultimate breach of the epithelial barrier, facilitating bacterial dissemination.

In conclusion, this study unveils a sophisticated virulence mechanism employed by *S. suis* to breach the host respiratory epithelial barrier. We propose a model in which the bacterial effector Stk1 interacts with the host protein Keap1, thereby enhancing the ubiquitination and proteasomal degradation of the master antioxidant regulator, Nrf2. The resulting collapse of the cellular antioxidant defense system sensitizes epithelial cells to ferroptosis, leading to the accumulation of lipid peroxides like 4-HNE. These lipid aldehydes, in turn, drive the upregulation of the transcriptional repressor Snail1, which suppresses the expression of key AJs and TJs protein. This cascade culminates in the loss of epithelial integrity, increased paracellular permeability, and ultimately, the facilitation of bacterial invasion and systemic dissemination (Figure 8). Existing studies have shown that various pathogenic bacteria can induce ferroptosis in host cells. For example, *M. tuberculosis* upregulates Bach1 to inhibit host antioxidant genes [21]; meanwhile, *P. aeruginosa* triggers ferroptosis by oxidizing phospholipids via pLoxA and degrading Gpx4 [22]. Based on this, it is hypothesized that ferroptosis-mediated damage to the epithelial barrier may be a common pathogenic strategy employed by multiple respiratory pathogens. By elucidating the complex interactions between bacterial effector proteins and the host ferroptosis pathway, this study deepens the understanding of the pathogenic mechanisms of *S. suis*. Further research confirms that the Nrf2-ferroptosis signalling axis can serve as a potential target for novel therapeutic interventions against this significant zoonotic pathogen.

## Materials and methods

### Bacterial strains and cell culture

The *S. suis* serotype 2 (SS2) strain SC19 and its isogenic mutant (SS2ΔStk1) and complemented (SS2CΔStk1) strains were routinely cultured at 37°C on Tryptic Soy Agar (TSA) plates or in Tryptic Soy Broth (TSB) medium (Zhejiang Tianhang Biotechnology Co., Ltd.), supplemented with 5% (v/v) heat-inactivated calf serum. Bacterial growth was monitored by measuring the optical density at 600 nm (OD600).

Porcine tracheal epithelial cells (NPTr) and human embryonic kidney cells (HEK293 T, ATCC CRL-3216) were maintained in Dulbecco’s Modified Eagle Medium (DMEM/High Glucose, Hyclone) supplemented with 10% (v/v) fetal bovine serum (FBS, SORFA) and 1% penicillin–streptomycin. Cells were cultured in a humidified incubator at 37°C with 5% CO2.

### Generation of knockout cell lines

To generate Snail1 and Keap1 knockout (KO) cell lines, single guide RNAs (sgRNAs) targeting the porcine SNAI1 and KEAP1 genes were designed and cloned into the lentiCRISPRv2 vector. sgRNA sequences are provided in Supplementary Table S1. Lentiviral particles were produced in HEK293 T cells and used to infect NPTr cells. After 48 h post-transduction, cells were selected with 10 µg/mL puromycin for another 72 h. Single-cell clones were isolated by limiting dilution, and the successful knockout of target proteins was confirmed by Western blot analysis.

### Antibodies and reagents

Primary antibodies used in this study were sourced as follows: rabbit anti-E-cadherin (#20874), rabbit anti-occludin (#27260), rabbit anti-Snail1 (#13099), mouse anti-GPX4 (#67763), rabbit anti-SLC7A11 (#26864), mouse anti-KEAP1 (#10503), rabbit anti-NRF2 (#16396), rabbit anti-ubiquitin (#10201), rabbit anti-HA tag (#51064), rabbit anti-FLAG tag (#20543), rabbit anti-Myc tag (#10828), and mouse anti-GFP tag (#50430) were purchased from Proteintech. Mouse anti-β-actin (#66009, Proteintech) was used as a loading control. Mouse anti-4-HNE (#MAB3249) was obtained from R&D Systems (Bio-Techne). Secondary antibodies, including Cy3-conjugated goat anti-mouse IgG (#SA00009) and FITC-conjugated goat anti-rabbit IgG (#80003), were from Proteintech. HRP-conjugated secondary antibodies were from Biodragon (#BF03008 and #BF03001). DAPI (#BL105B) was purchased from Biosharp.

The chemical inhibitors and agonists used were: RSL3 (#HY-100218A), Ferrostatin-1 (#HY-100579), MG132 (#HY-13259), and chloroquine (CQ, #HY-17589) from MedChemExpress (MCE), and 4-HNE (#S9793) from Selleck Chemicals.

### Plasmid construction and transfection

For protein expression, genes encoding SS2 virulence factors (Sly, Stk1, Cps2c, Eno, Mrp, DnaK, Dpp4, Ef, GdhA, VicK, ApdS) and porcine Nrf2 and Keap1 were PCR-amplified and cloned into various mammalian expression vectors, including pCAGGS-HA, pCMV14-3xFlag, pCMV14-Myc, and pEGFP-C1, as specified for each experiment. All primers used for plasmid construction are listed in Supplementary Table S2. Plasmids were transfected into NPTr or HEK293 T cells using the jetPRIME® transfection reagent (Polyplus) according to the manufacturer’s protocol.

### Animal experiments

All animal experiments were conducted in strict accordance with the guidelines of the Ministry of Science and Technology of the People’s Republic of China for Laboratory Animal Care and Use and were approved by the Hubei Provincial Laboratory Animal Management Committee (Piglets: HZAUSW-2024-0084; Mice: HZAUMO-2024-0301).

Three- to four-week-old healthy Large White piglets (n = 3 per group) were sourced from a commercial breeding company in Hubei Province and were intratracheally inoculated with 1 × 10^10^ CFU of SS2 SC19 or sterile PBS. At 36 h post-infection (hpi), piglets were euthanized, and tracheal and lung tissues were harvested for bacterial load determination, histology, and molecular analyses.

Three- to four-week-old female specific-pathogen-free (SPF) Kunming mice (n = 10 per group) were obtained from the Laboratory Animal Center of Huazhong Agricultural University. Mice were intratracheally inoculated with 6 × 10^8^ CFU of SS2, SS2ΔStk1, SS2CΔStk1, or PBS. For inhibitor studies, a separate cohort of mice was treated with Ferrostatin-1 (10 mg/kg) via intraperitoneal injection 1 h prior to infection, followed by a second dose (1 mg/kg) via tail vein injection at 6 hpi. At 18 hpi, mice were euthanized, and blood, tracheal, and lung tissues were collected for downstream analysis.

### Measurement of transepithelial electrical resistance (TEER)

The integrity of the NPTr cell monolayer was monitored in real-time by measuring TEER using two complementary methods. For continuous monitoring, cells were seeded on collagen-coated gold electrodes (96W1E + array, Applied BioPhysics) and measured using an ECIS Z-Theta system (Applied BioPhysics), as previously described [57]. For endpoint measurements, cells were cultured on Transwell inserts (0.4 µm pore size, Corning) until a stable resistance was achieved. Resistance across the monolayer was measured using an EVOM2 epithelial voltohmmeter (World Precision Instruments). After stabilization, cells were treated with SS2, 4-HNE, or RSL3 in the apical chamber, and TEER values were recorded at indicated time points.

### Cell viability and death assays

Cell viability was quantified using the Cell Counting Kit-8 (CCK-8) assay (Beyotime). Briefly, 1 × 104 cells/well were seeded in 96-well plates. Following treatment, 10 µL of CCK-8 solution was added to each well, and plates were incubated for 1 h at 37°C. Absorbance at 450 nm was measured using a Spark multimode microplate reader (Tecan). Cell death was assessed by propidium iodide (PI) staining (Beyotime). Following treatment, cells were fixed with 4% paraformaldehyde and then stained with PI in the dark for 10 min, as per the manufacturer’s instructions. PI fluorescence was quantified using the Spark microplate reader or visualized at 450 nm using a fluorescence microscope (Sage Science).

### Transmission electron microscopy (TEM)

NPTr cells were fixed with 2.5% glutaraldehyde in 0.1 M cacodylate buffer (pH 7.4) for 1 h, post-fixed with 1% osmium tetroxide, and stained with 2% uranyl acetate. Samples were then dehydrated in a graded ethanol series and embedded in Embed-812 resin (Electron Microscopy Sciences). Ultrathin (60 nm) sections were cut using a Leica ultramicrotome, placed on carbon-coated grids, and contrasted with 2% uranyl acetate and lead citrate. Grids were examined using a Tecnai G2 20Twin electron microscope (FEI).

### Biochemical assays

Intracellular levels of ferrous iron (Fe^2+^), glutathione (GSH), and malondialdehyde (MDA) were quantified using commercially available colorimetric assay kits in accordance with the manufacturers’ instructions. Briefly, treated NPTr cells were lysed on ice for 10 min and then centrifuged at 15,000×g for 10 min to collect the supernatant. The intracellular Fe^2+^ concentration was determined using the Cell Ferrous Ion Colorimetric Assay Kit (Elabscience, E-BC-K881-M), and absorbance was measured at 593 nm with a Spark multi-functional microplate reader (Tecan).

Intracellular GSH levels were assessed using the GSH and GSSG Assay Kit (Beyotime, #S0053). Following treatment, cell samples underwent two rapid freeze–thaw cycles using liquid nitrogen and a 37°C water bath. After centrifugation, the supernatant was used for analysis. According to the manufacturer’s protocol, the supernatant, GSH clarifying solution, and total GSH assay working solution were added sequentially. The reaction mixture was incubated at 25°C for 1 h, followed by the addition of NADPH (0.5 mg/mL) and further incubation at 25°C for 25 min. Absorbance was measured at 405 nm using the Spark microplate reader to determine GSH concentration.

Lipid peroxidation was evaluated by measuring MDA levels via its reaction with thiobarbituric acid (TBA), which forms a red chromogenic product. Using the Lipid Peroxidation MDA Assay Kit (Solarbio, #BC0025), cell lysates from different treatment groups were mixed with MDA detection reagent, heated at 100°C for 1 h, cooled on ice, and centrifuged at 10,000×g for 10 min. The resulting supernatant was collected, and absorbance was recorded at 532 and 600 nm using the Spark multi-functional microplate reader to calculate MDA content.

### Measurement of ROS and lipid peroxidation

Intracellular reactive oxygen species (ROS) were measured using the DCFH-DA probe (Beyotime, #S0033S). Treated NPTr cells were incubated with 5 µM DCFH-DA in the dark for 20 min at 37°C. The fluorescence of the oxidized product, DCF, was analysed by flow cytometry (Beckman Coulter).

Lipid peroxidation was assessed using the fluorescent probe C11-BIODIY 581/591 (Sigma, #SML3717). Cells were incubated with 5 µM C11-BIODIY for 1 h at 37°C. The fluorescence shift indicating lipid peroxidation was quantified using the Spark microplate reader or visualized by confocal microscopy (Nikon).

### Histology and immunostaining

For immunofluorescence assays (IFA), cells grown on coverslips or paraffin-embedded tissue sections were fixed with 4% paraformaldehyde for 10 min and permeabilized with 0.3% Triton X-100 for 15 min. After blocking with 5% BSA, samples were incubated with primary antibodies overnight at 4°C, followed by incubation with appropriate fluorescently-conjugated secondary antibodies for 1 h. Nuclei were counterstained with DAPI. Images were captured using a Nikon confocal microscope.

For histological analysis, paraffin-embedded tracheal and lung tissues were sectioned, deparaffinized, and stained with haematoxylin and eosin (H&E). For immunohistochemistry (IHC), tissue sections were rehydrated through a graded ethanol series and rinsed with distilled water. Antigen retrieval was performed using established protocols. Sections were subsequently washed with PBS and incubated with a blocking solution containing 3% BSA for 30 min at room temperature. The primary antibody against 4-HNE was applied at an appropriate dilution and incubated overnight at 4°C. The sections were incubated with horseradish peroxidase (HRP)-conjugated goat anti-mouse secondary antibody at room temperature for 50 min in the dark. After washing with PBS, the sections were incubated with DAB chromogenic solution. Subsequently, the sections underwent tissue clearing and were mounted with a coverslip. Finally, representative images were captured using a light microscope.

### Western blotting and co-Immunoprecipitation (Co-IP)

Cells and homogenized tissues were lysed in RIPA buffer (MedChemExpress, HY-K1001) supplemented with a protease inhibitor cocktail (MedChemExpress, HY-K0010). Protein concentrations were determined using a BCA protein assay kit. Equal amounts of protein were mixed with loading buffer, denatured by heating at 100°C for 10 min, and subsequently separated via SDS-PAGE. Proteins were then transferred onto PVDF membranes (Merck Millipore, IPVH00010), blocked with 5% skimmed milk in Tris-buffered saline containing Tween-20 (TBST), and incubated overnight at 4°C with primary antibodies. Following stringent washing steps, the membranes were incubated with HRP-conjugated secondary antibodies. Chemiluminescent signals were visualized using a ChemiDoc Imaging System (Bio-Rad).

For Co-IP, cells were lysed in IP lysis buffer (Biosharp, BL509A) and incubated overnight at 4°C with anti-NRF2 antibody or anti-Flag magnetic beads (MCE, HY-K0207). The resulting immune complexes were captured with Protein A/G agarose beads (for primary antibody IP) or by magnetic separation. After extensive washing, bound proteins were eluted by boiling in SDS-PAGE loading buffer and analysed further by Western blot.

### RNA sequencing and bioinformatic analysis

Total RNA was extracted from NPTr cells (uninfected or SS2-infected for 6 h) using TRIzol reagent (Invitrogen). RNA integrity was assessed using an Agilent 2100 Bioanalyzer. Library preparation and paired-end 150 bp sequencing were performed on the MGI-2000 platform by BGI Genomics. Raw sequencing reads were quality-filtered using SOAPnuke (v2.1.0) to remove adapter sequences and low-quality reads. The clean reads were then aligned to the pig reference genome (Sscrofa11.1) using HISAT2 (v2.1.0). Gene expression levels were quantified as fragments per kilobase of transcript per million mapped reads (FPKM). Differentially expressed genes (DEGs) were identified using DESeq2 (v1.22.2) with a false discovery rate (FDR) < 0.05 and an absolute log2 fold change > 1. Gene Ontology (GO) and Kyoto Encyclopedia of Genes and Genomes (KEGG) pathway enrichment analyses were performed on the DEG list. The RNA-seq data have been deposited in the NCBI Gene Expression Omnibus (GEO) (accession number: GSE311550).

### Quantitative real-Time PCR (qRT-PCR)

Total RNA was reverse-transcribed into cDNA using the HiScript III RT SuperMix for qPCR (Vazyme, #R223). qRT-PCR was performed using ChamQ Universal SYBR qPCR Master Mix (Vazyme, #Q312) on a QuantStudio 3 Real-Time PCR System (Applied Biosystems). The relative expression of target genes was calculated using the 2^−ΔΔCt^ method, with β-actin serving as the endogenous control. All primer sequences are listed in Supplementary Table S3.

### Statistical analysis

Data are presented as the mean ± standard deviation (SD) from at least three independent experiments. Statistical analyses were performed using GraphPad Prism (v10.1.2). Statistical significance was analysed by one-way ANOVA test. *p*-value < 0.05 was considered statistically significant (*p < 0.05, **p < 0.01, ***p < 0.001, ns, no significant).

## Supplementary Material

Supplemental Material

Supporting Information Clean 20260130.docx

FigureS1.jpg

FigureS2.jpg

FigureS3.jpg

FigureS5.jpg

## Data Availability

The data that support the findings of this study are available in the supplementary material of this article.
