# Supplementary figures and images for: Streptococcus suis Stk1 sensitizes epithelial cells to ferroptosis and exacerbates disruption of the respiratory epithelial barrier
Source: Emerg Microbes Infect. 2026 Feb 2;15(1):2627066. doi: 10.1080/22221751.2026.2627066 (PMC12927416; doi:10.1080/22221751.2026.2627066)

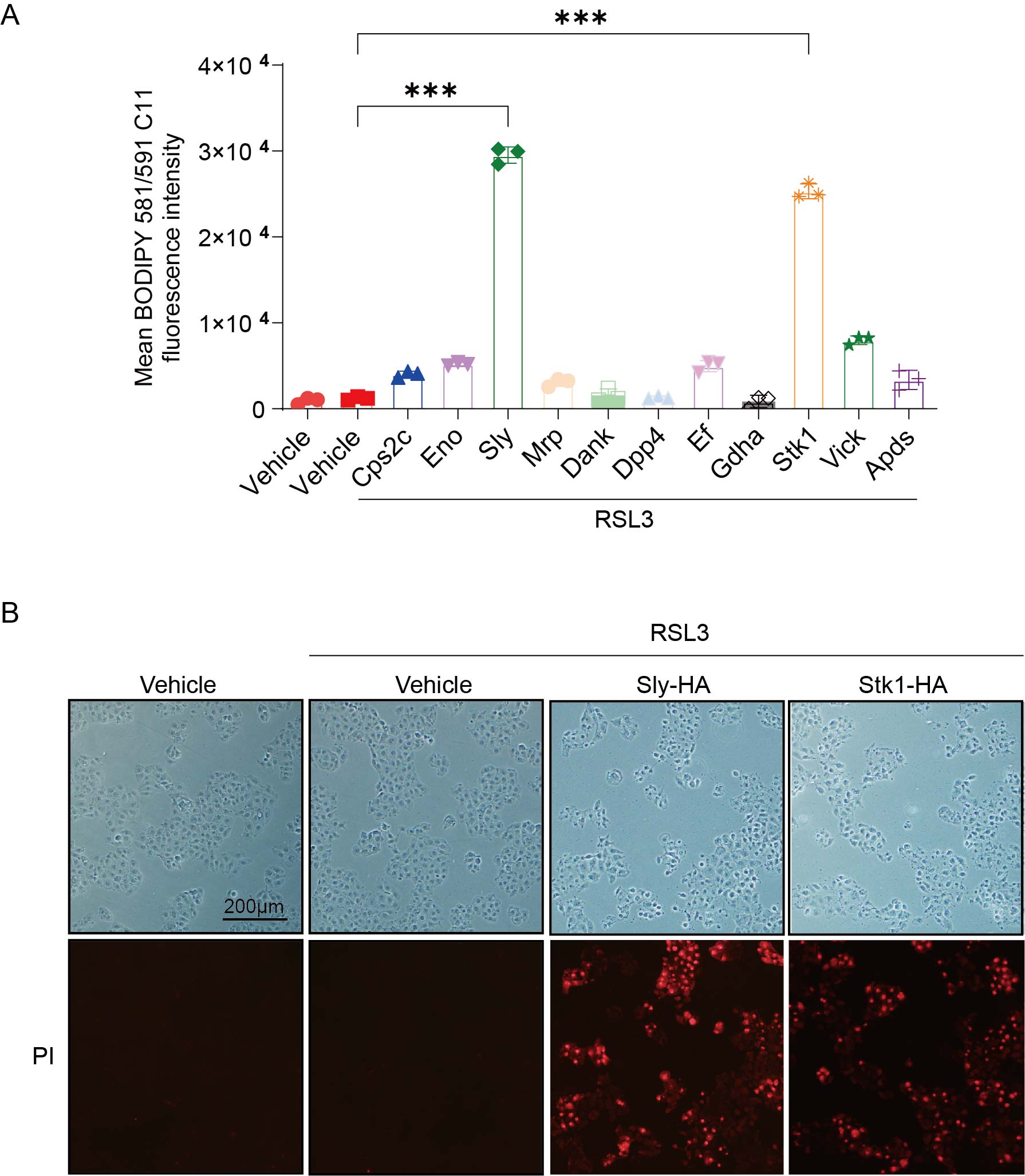

Supplement: Supplemental Material [file TEMI_A_2627066_SM0822.jpg]

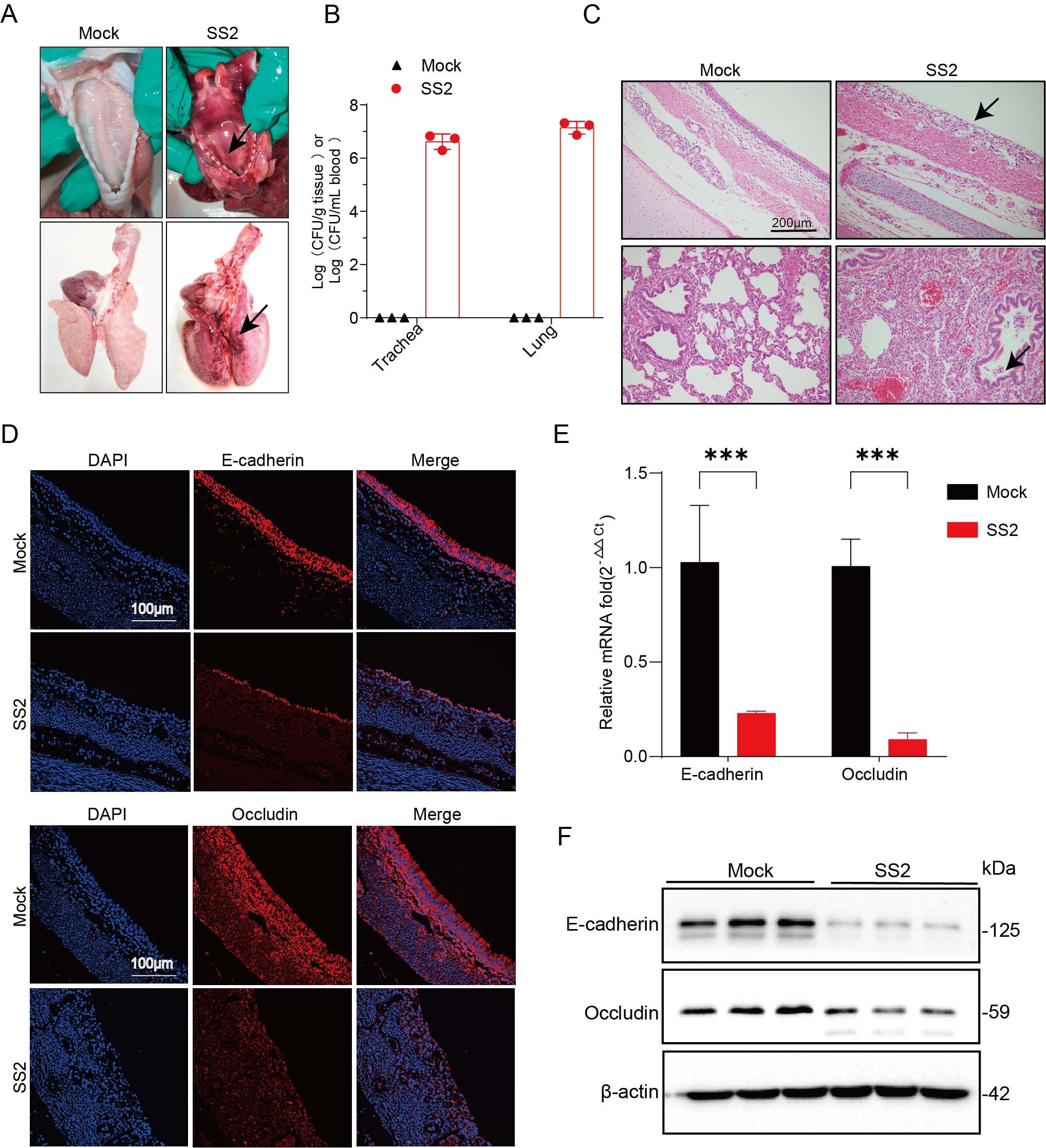

Supplement: FigureS1.jpg [file TEMI_A_2627066_SM2043.jpg]

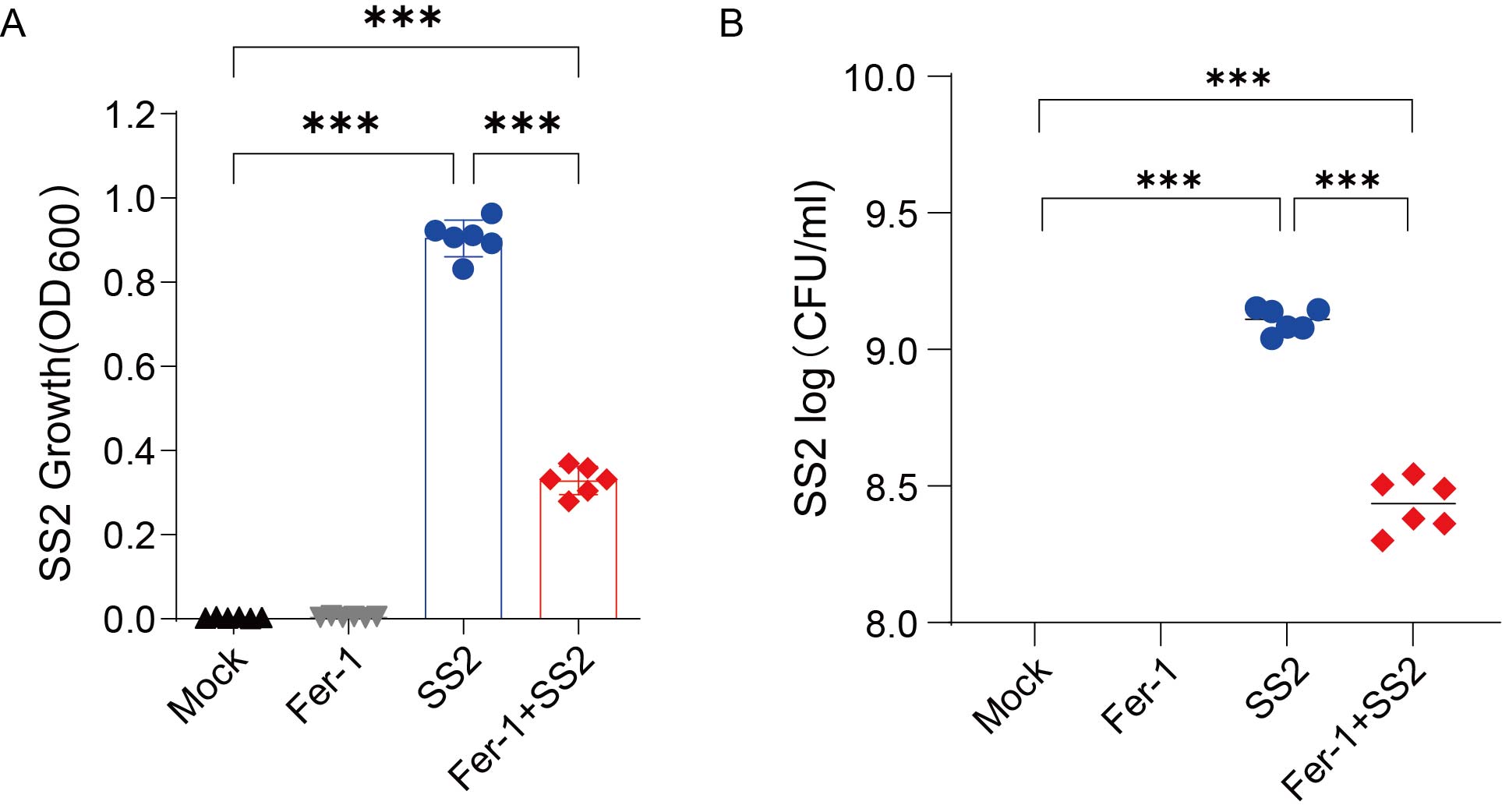

Supplement: FigureS2.jpg [file TEMI_A_2627066_SM2042.jpg]

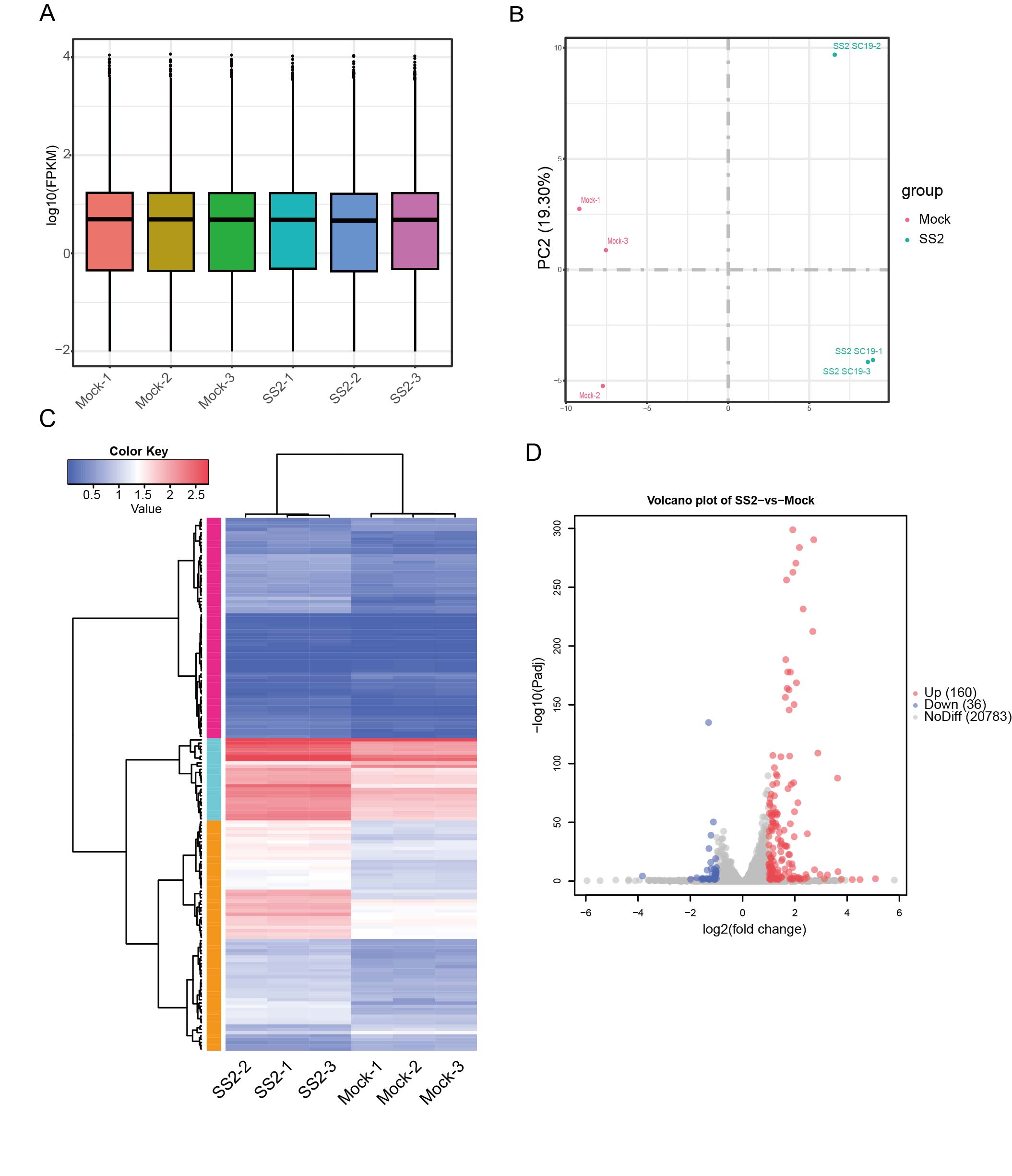

Supplement: FigureS3.jpg [file TEMI_A_2627066_SM2041.jpg]

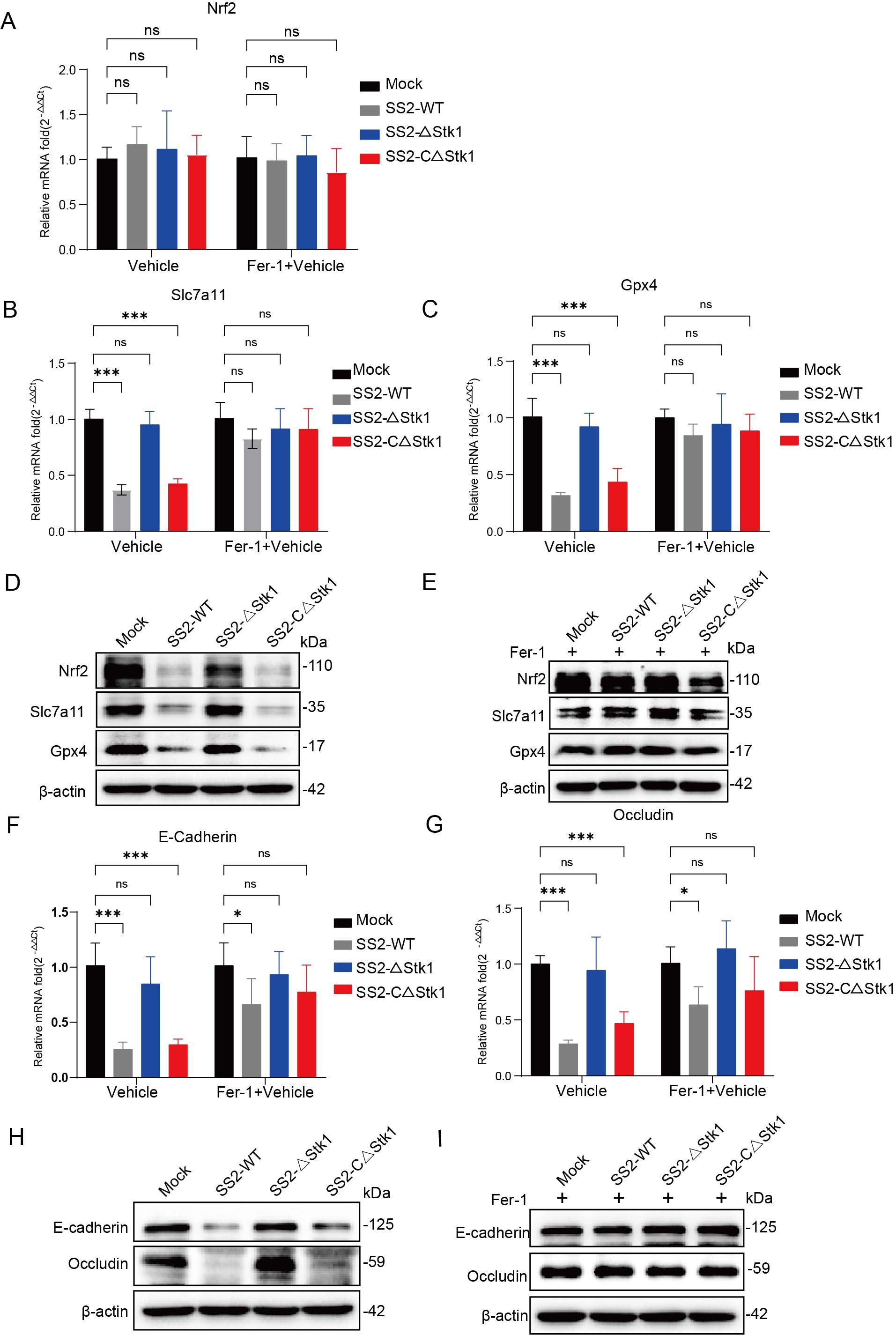

Supplement: FigureS5.jpg [file TEMI_A_2627066_SM2040.jpg]
