# Supplementary material for: Streptococcus suis Stk1 sensitizes epithelial cells to ferroptosis and exacerbates disruption of the respiratory epithelial barrier
Source: Emerg Microbes Infect. 2026 Feb 2;15(1):2627066. doi: 10.1080/22221751.2026.2627066 (PMC12927416; doi:10.1080/22221751.2026.2627066)
Supplement: Supporting Information Clean 20260130.docx [file TEMI_A_2627066_SM2044.docx]

# **Supporting Information**

**Streptococcus suis Stk1 Sensitizes Epithelial Cells to Ferroptosis and Exacerbates Disruption of the Respiratory Epithelial Barrier**

Lang Tian, Ruicheng Yang, Ting Qi, Wenquan Ouyang, Hongshuo Liu, Dong Huo, Hang Li, Chuyue Zhou, Manman Xu, Haojie Li, Qingyun Liu, Dang Wang, Chen Tan, Huanchun Chen, Xiangru Wang ^*^

**Supplementary Figure Legends**

**
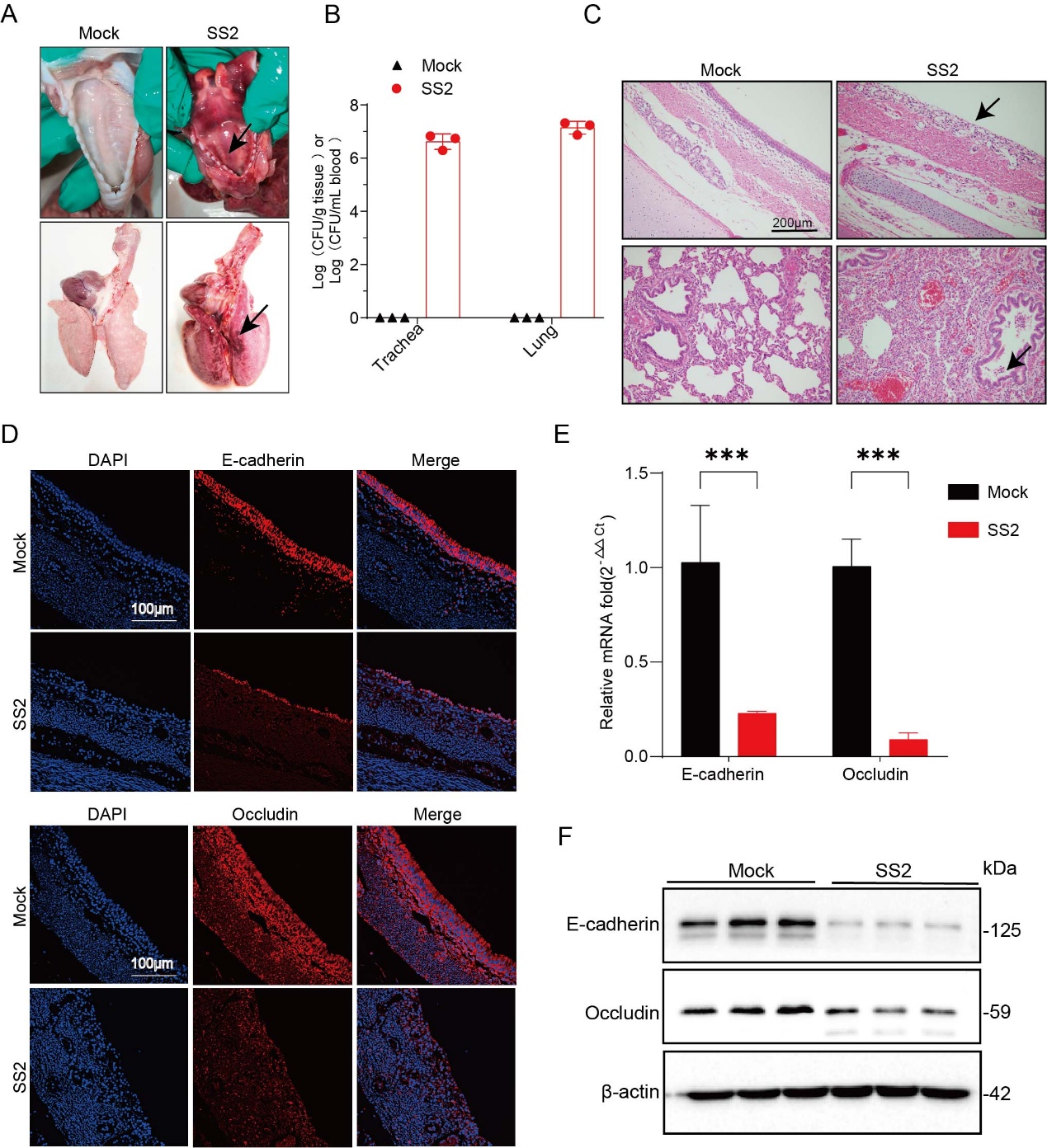
**

**Figure S1. *S. suis* infection disrupts the respiratory epithelial barrier in a piglet model.**

(A-F) Piglets were intratracheally infected with SS2 (1×10^10^ CFU) or PBS for 36 hours. (A) Gross pathology of the trachea and lungs. (B) Bacterial loads in the trachea and lungs. (C) Representative H&E-stained sections of tracheal and lung tissues. Scale bar, 200 µm. (D) Immunofluorescence analysis of E-cadherin and Occludin in piglet tracheal tissue using confocal microscopy. Scale bar, 100 µm. (E) qRT-PCR analysis of E-cadherin and Occludin mRNA levels in tracheal tissue. (F) Western blot analysis of E-cadherin and Occludin protein levels in tracheal tissue. Data are presented as mean ± SD (n=3 piglets per group). Statistical significance was determined by one-way ANOVA test for other panels (**p* < 0.05; ***p* < 0.01; ****p* < 0.001).

**
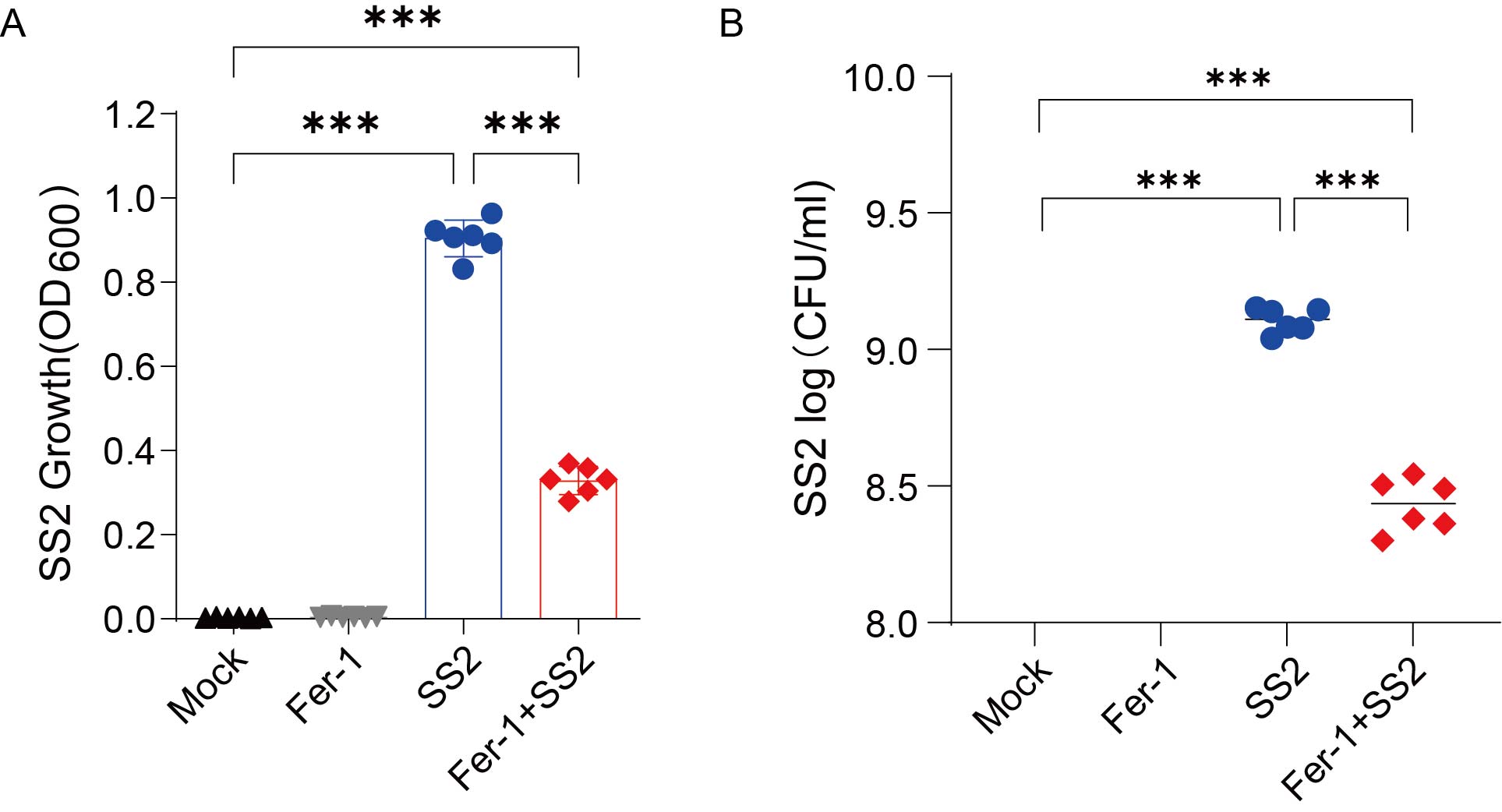
**

**Figure S2. Inhibition of ferroptosis restricts the proliferation of *S. suis*.**

(A-B) NPTr cells were pretreated with Fer-1 (20 µM) or vehicle for 2 hours before infection with SS2 (MOI=10) for 6 hours. Bacterial proliferation was assessed by (A) measuring OD600 of lysed cell cultures and (B) direct colony counting (CFU/mL). Data are presented as mean ± SD from three independent experiments. Statistical significance was determined by one-way ANOVA test for other panels (**p* < 0.05; ***p* < 0.01; ****p* < 0.001).


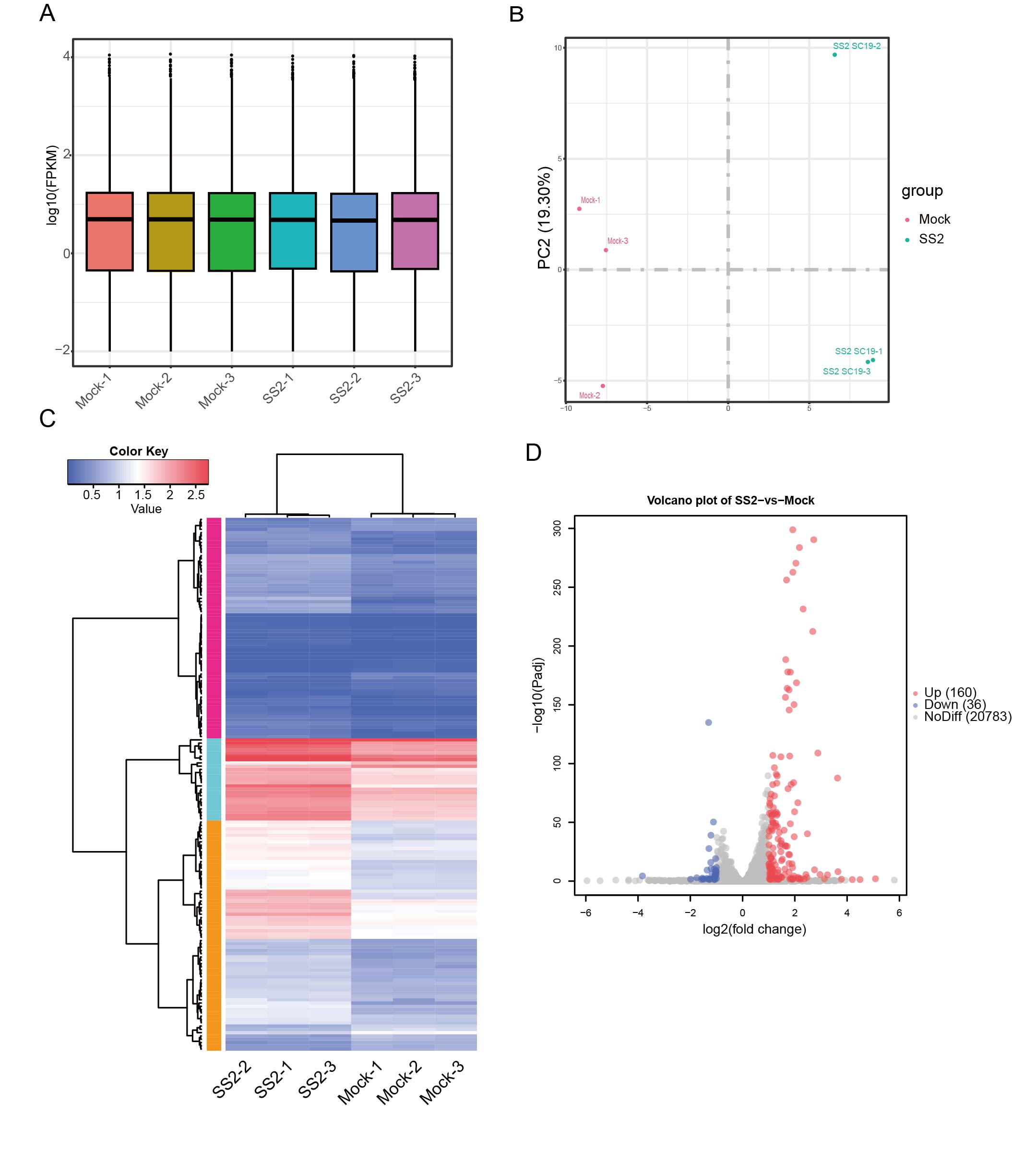


**Figure S3. Transcriptomic profiling of SS2-infected respiratory epithelial cells.**

(A-D) NPTr cells were infected with SS2 (MOI=10) or treated with PBS for 6 hours, followed by RNA sequencing. (A) FPKM density distribution plots comparing global gene expression. (B) Principal component analysis (PCA) plot showing distinct clustering of control and SS2-infected samples. (C) Hierarchical clustering heatmap and (D) volcano plot analysis of differentially expressed genes (DEGs).


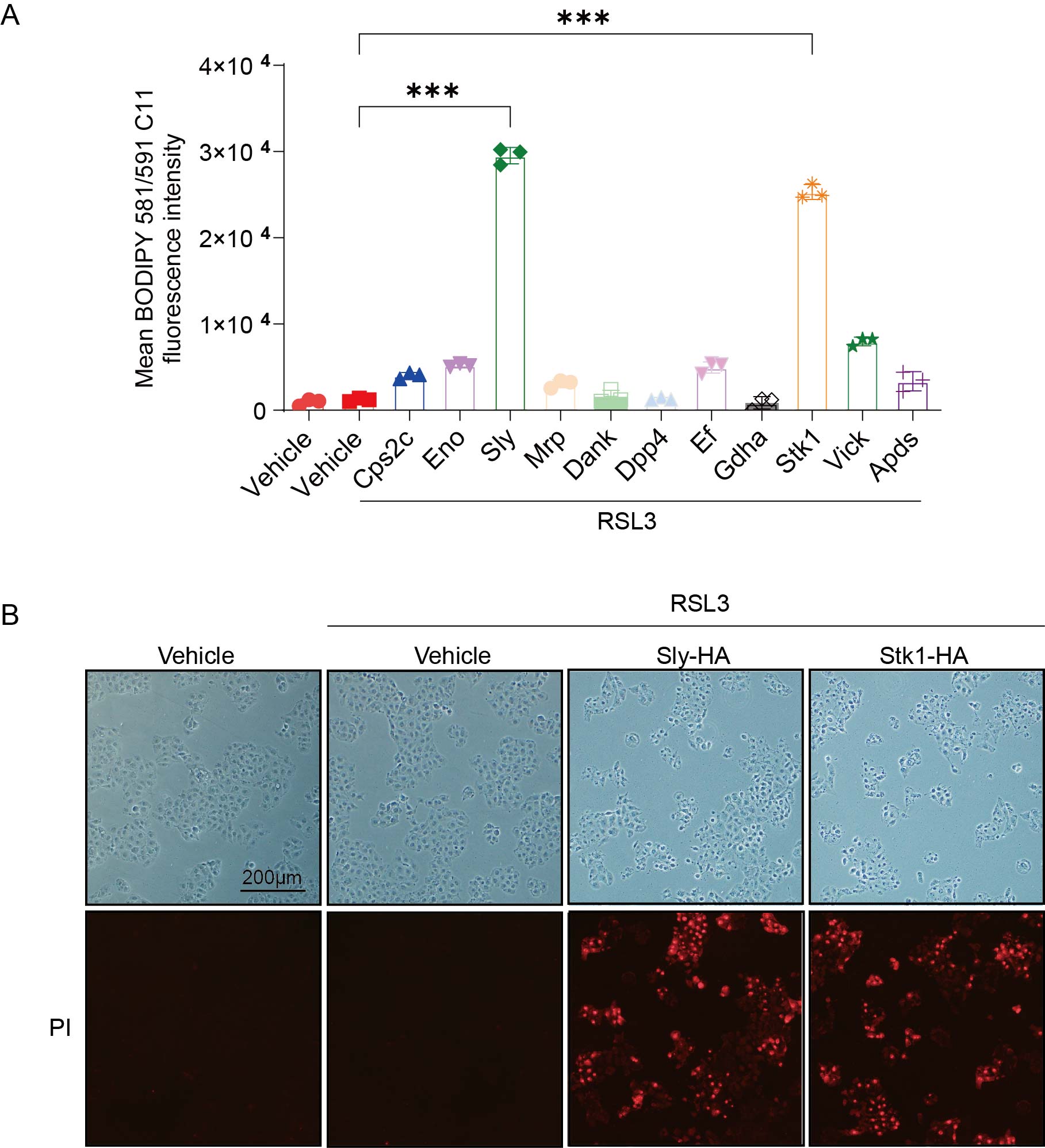


**Figure S4. Screening of SS2 virulence factors involved in sensitizing cells to ferroptosis.**

(A) NPTr cells were transfected with plasmids expressing the indicated GFP-tagged SS2 virulence factors for 24 hours, then treated with RSL3 (100 nM) for an additional 24 hours. Lipid peroxidation was quantified by C11-BODIPY fluorescence. (B) NPTr cells were transfected with plasmids expressing Sly-HA or Stk1-HA, followed by RSL3 treatment. Cell death was visualized by PI staining. Scale bar, 200 µm. Data are presented as mean ± SD from three independent experiments. Statistical significance was determined by one-way ANOVA test for other panels (**p* < 0.05; ***p* < 0.01; ****p* < 0.001).

**
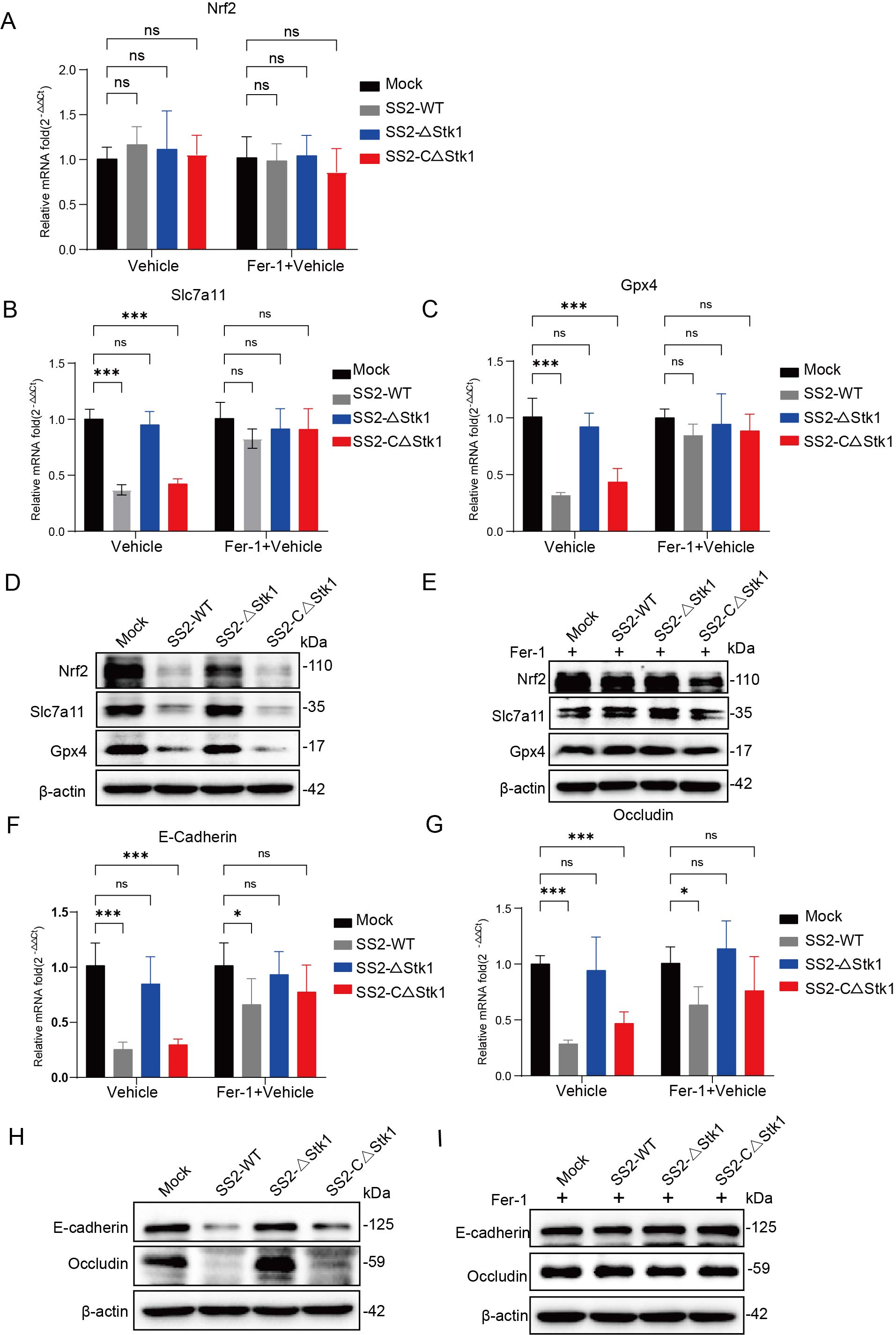
**

**Figure S5. Stk1 modulates the Nrf2 antioxidant pathway and junctional protein expression in vivo.**

Mice were intratracheally infected with 6 × 10^8^ CFU of SS2 WT, ΔStk1, or CΔStk1 and treated with Fer-1 or vehicle. Tracheal tissues were collected at 18 hpi. (A-C) qRT-PCR analysis of Nrf2, Slc7a11, and Gpx4 mRNA levels in tracheal tissue. (D-E) Western blot analysis of Nrf2, Slc7a11, and Gpx4 protein levels in tracheal tissue. (F-G) qRT-PCR analysis of E-cadherin and Occludin mRNA levels in tracheal tissue. (H-I) Western blot analysis of E-cadherin and Occludin protein levels in tracheal tissue. Data are presented as mean ± SD from three independent experiments (n=5 mice per group). Statistical significance was determined by one-way ANOVA test for other panels (**p* < 0.05; ***p* < 0.01; ****p* < 0.001, ns, no significant).

**Table S1.**

**Guide RNA（gRNA）Sequence were used in this study.**

| Gene name | Guide RNA（gRNA） sequence |
| --- | --- |
| Snail1-KO | sgRNA1：CAACAAGGAGTATGTCAGCCTGG |
|  | sgRNA2：CAATTGGCCCAAGCCTGGGAAGG |
| Keap1-KO | sgRNA1：CGTACCACGAAATCGAATTGGGG |
|  | sgRNA2：GGCCGGAACAATTCACCCGACGG |

**Table S2.**

**Primers used in this study for PCR.**

| Primer name | Sequence (5'–3') |
| --- | --- |
| Cps2c-GFP | F: GATCTCGAGATATGGAAAAAGTCAGCATTATTGTACC |
|  | R: GGTGGATCCTTAATCATTATTTTTTTCTTCCC |
| Eno-GFP | F: GATCTCGAGATATGTCAATTATTACTGATGTTTAC |
|  | R: GGTGGATCCTTATTTTTTCAAGTTGTAGAATG |
| Sly-GFP | F: GATCTCGAGATATGAGAAAAAGTTCGCACTTGATTTTAAGC |
|  | R: GGTGGATCCTTACTCTATCACCTCATCCGCAT |
| Sly-HA | F: GCTGAATTCATGAGAAAAAGTTCGCACTTGATTTTAAGC |
|  | R: GCTGAATTCTTACTCTATCACCTCATCCGCAT |
| Mrp-GFP | F: GATCTCGAGATATGCGTAGATCAAATAAAAAATC |
|  | R: GGTGGATCCCTAATCTTCGTTACGACGACGTTTTC |
| Dank-GFP | F: GATCTCGAGATATGTCTAAAATTATCGGTATTGAC |
|  | R: GGTGGATCCCTACTTTTCAGTAAACTCGCCATC |
| Dpp4-GFP | F: GATCTCGAGATATGCGCTTTAATCAATTTTCTTTC |
|  | R: GGTGGATCCTTAGTGCGGTAAAATTAACTGTG |
| Ef-GFP | F: GATCTCGAGATATGTCTTATAAAGATATGTTCAG |
|  | R: GGTGGATCCCTACGCCTTTGTCCTCTGCCGA |
| Gdha GFP | F: GATCTCGAGATATGTCAAATGCCAAAGCTTACATCC |
|  | R: GGTGGATCCTTATACCAAACCTTGGGCAATCATGC |
| Stk1-GFP | F: GATCTCGAGATATGATTCAAATCGGTAAGATCTTTG |
|  | R: GGTGGATCCTTATTGTCCGCTACCTGTTGAAG |
| Stk1-HA | F: GCTGAATTCATGATTCAAATCGGTAAGATCTTTGC |
|  | R: GCTGAATTCTTATTGTCCGCTACCTGTTGAAG |
| Vick-GFP | F: GATCTCGAGATATGATTAATCAATTACGTTATTTAATG |
|  | R: GGTGGATCCCTATTCTTCCTCATCTGAATCAT |
| Apds-HA | F: GATCTCGAGATATGAAAAAGACTTTTGTTCGTATAG |
|  | R: GGTGGATCCTTATTCTTTACTGGATTTTTTTCGATG |
| Stk1-Flag | F: CGCGAATTCATGATTCAAATCGGTAAGATCTTTGC |
|  | R: CCGGGATCCTTGTCCGCTACCTGTTGAAGTAC |
| Nrf2-HA | F: GCTGAATTCATGACCCCTCTGGGCCGTCTAA |
|  | R: GCTGAATTCCTAGTTTTTCTTCACATCTGGCTTCT |
| Nrf2-Flag | F: CGCGAATTCATGACCCCTCTGGGCCGTCTAA |
|  | R: CCGGGATCCCTAGTTTTTCTTCACATCTGGCTTCT |
| Keap1-HA | F: GCTGAATTCCTAGTTTTTCTTCACATCTGGCTTCT |
|  | R: GCTGAATTCCTAGTTTTTCTTCACATCTGGCTTCT |
| Keap1- Flag | F: CGCGAATTCCTAGTTTTTCTTCACATCTGGCTTCT |
|  | R: CCGGGATCCCTAGTTTTTCTTCACATCTGGCTTCT |
| Keap1- Myc | F: CCCGAATTCCTAGTTTTTCTTCACATCTGGCTTCT |
|  | R: TACCTCGAGCTAGTTTTTCTTCACATCTGGCTTCT |

**Table S3.**

**Primers used in this study for qRT-PCR.**

| Primer name | Sequence (5'–3') |
| --- | --- |
| Occludin-Sus scrofa | F: GCTTTCTCAGCCAGCGTATT |
|  | R: GGGTAGCCCATACCACCTCC |
| Occludin-Mouse | F: GCCTTCTGCTTCATCGCTTC |
|  | R: CGTCGGGTTCACTCCCATTA |
| E-cadherin-Sus scrofa | F: TCTCTACTTCCACGGCCACT |
|  | R: GATGTGATCTCCAGGCCCAC |
| E-cadherin-Mouse | F: GGACGTCCATGTGTGTGACT |
|  | R: GATCAGAATCAGCAGGGCGA |
| β-actin-Sus scrofa | F: TGCGGGACATCAAGGAGAAG |
|  | R: AGTTGAAGGTGGTCTCGTGG |
| β-actin-Mouse | F: GAGAAGCTGTGCTATGTTGCT |
|  | R: AAAGAGCCTCAGGGCATCG |
| Nrf2-Sus scrofa | F: GCGAGGTAATCCGTCCATCC |
|  | R: GTTCAGTCGCTTCACGTCGG |
| Nrf2-Mouse | F: AACAGAACGGCCCTAAAGCA |
|  | R: TGGGATTCACGCATAGGAGC |
| Slc7a11-Sus scrofa | F: CTGGGCAGGAGAAAGTTGTG |
|  | R: CATACGGTCCAGACGACCA |
| Slc7a11-Mouse | F: TGCTGGCTGGTTTTACCTCA |
|  | R: AGCCCACTGTGATGATAGCC |
| Gpx4-Sus scrofa | F: TGAGCTTTAGCCGCCTGTTC |
|  | R: CCTTGGCTGAGAATTCGTGC |
| Gpx4-Mouse | F: CTAGTCGATCTGCATGCCCG |
|  | R: GGCATCGTCCCCATTTACAC |
